# Developing Multi-Component Solid Formulation Strategies for PROTAC Dissolution Enhancement

**DOI:** 10.1021/acs.molpharmaceut.5c01107

**Published:** 2025-10-02

**Authors:** Martin A. Screen, Sean Askin, James F. McCabe, Esther Jacobs, Akosua Anane-Adjei, Clare S. Mahon, Mark R. Wilson, Jonathan W. Steed

**Affiliations:** † Department of Chemistry, 3057 Durham University, South Road, Durham DH1 3LE, U.K.; ‡ Advanced Drug Delivery, Pharmaceutical Sciences, R&D, AstraZeneca, Cambridge CB2 0AA, U.K.; § Early Pharmaceutical Development & Manufacture, Pharmaceutical Sciences, R&D, AstraZeneca, Macclesfield SK10 2NA, U.K.

**Keywords:** PROTACs, amorphous, ASD, dissolution, formulation

## Abstract

PROTACs are an emerging class of beyond-rule-of-5 molecular drugs currently under clinical investigation for the treatment of malignant diseases and are capable of degrading previously “undruggable” protein targets. They are poorly crystallizable due to their structure, consisting of two ligands joined chemically by a flexible linker, yet the inherent insolubility of their amorphous phases hinders their development into sufficiently bioavailable medicines. Formulation approaches to improve the dissolution properties of PROTACs are required as a result, but research in this area is made even more challenging by the scarcity of available samples. In this work, amorphous solid dispersion (ASD) formulations of four cereblon-recruiting PROTACs ‘AZ1–4’ using hydroxypropyl methylcellulose acetate succinate (HPMCAS) as a polymer excipient are described. ASDs of AZ1 show up to a 2-fold increase in drug supersaturation compared to the pure amorphous API, observed up to a drug loading of 20% w/w. Preparing the ASDs by slurry conversion offers greater solubility enhancement over those prepared by solvent evaporation and maintains the dissolution advantage up to a higher drug load. Positive deviations from theoretical *T*_g_ values coupled with a lack of spectral evidence of drug-polymer hydrogen-bond interactions suggest that the ASDs may differ from ideal mixtures via predominantly dispersive drug-polymer interactions. ASDs that provide a dissolution enhancement were stored at elevated temperature and humidity for one month and showed no sign of plasticization or loss of physical stability. Coamorphous formulations using low-molecular-weight excipients, by contrast, showed no dissolution advantage despite evidence of drug–coformer hydrogen-bonding interactions. This work demonstrates that ASDs may be an effective strategy for improving PROTAC bioavailability and producing commercializable solid forms for oral administration despite the lack of well-behaved solid phases of PROTACs. It also highlights the need for a deeper understanding of how to develop successful formulation approaches for bRo5 compounds.

## Graphical abstract

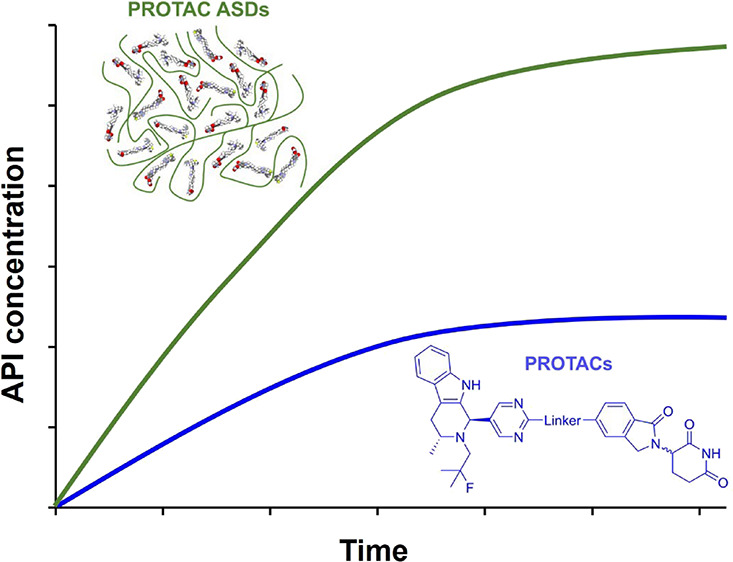

## Introduction

Proteolysis targeting chimeras (PROTACs) are an emerging class of molecular drugs in a chemical space beyond Lipinski’s rule of 5 (bRo5),^1^ designed to degrade target disease-causing proteins previously considered “undruggable” by conventional small molecule drugs. Unlike traditional small molecule inhibitors, PROTACs are capable of degrading a protein by harnessing the ubiquitin-proteosome system, rather than only blocking its enzymatic function by binding to the enzymatic site.^2^ This affords PROTACs the benefits of lower dosing potential, since they act via an event-driven mechanism and do not have to remain bound to the protein of interest to have a therapeutic effect. However, their large size and structural complexity present challenges regarding their cell permeability, solubility, and pharmacokinetic properties. Very poor aqueous solubility impedes their development into drug products with sufficient oral bioavailability,^3^ and with numerous ionizable atoms, the conventional processes of pharmaceutical salt and cocrystal screening are made much more complicated.^4^

Multicomponent solids, which can offer advantageous chemical and physical characteristics compared to a pure active pharmaceutical ingredient (API) without modifying its chemical structure or pharmacology,^5–8^ usually consist of two molecular components bonded by noncovalent interactions such as hydrogen-bonding, π–π stacking, van der Waals forces or halogen-bonding, and as such they are often designed using an understanding of the possible supramolecular motifs that can form between the two components.^5^ The resulting solids can be either crystalline or amorphous. The latter possess no long-range order but often show improved solubility and/or dissolution rate compared to their cocrystalline counterpart through their higher free energy.^8–10^ Compared to an inherently unstable pure amorphous drug, coamorphous formulations are often less likely to recrystallize into the crystalline drug form and lose their dissolution advantage.^11^ The glass transition temperature (*T*_g_) of the resulting amorphous solid is usually between that of the two compounds, which would suggest greater molecular mobility and lower kinetic stability than the single component with the higher *T*_g_; however, the coamorphous phase is usually further physically stabilized by intermolecular interactions between components. This is in part because the formation of strong heterodimer interactions can disrupt short-range molecular ordering of one component, where the formation of homodimers often precedes recrystallization and the loss of the dissolution advantage provided by the coamorphous phase.^12^ Strong intermolecular interactions between components in a coamorphous solid can usually be identified by the *T*_g_ value deviating from the theoretical *T*_g_ calculated via the Gordon–Taylor equation,^13–16^ which predicts the *T*_g_ of a homogeneous coamorphous solid assuming no specific interactions between components (ideal mixing) and ideal free volume additivity of the two components.^12^ The greater physical stability that they impart allows the more soluble and faster dissolving amorphous solid to persist for a longer duration in contact with solution, maintaining supersaturation of the API for a longer duration before recrystallization. This type of dissolution kinetics is often referred to as a “spring and parachute.” In some systems, such as coamorphous naproxen/cimetidine, heterodimer formation involving hydrogen bonds has also been shown to cause synchronous dissolution of the individual components, as well as enhancing the dissolution rate.^17^ Hence, producing a coamorphous formulation involving strong noncovalent bonds between a poorly soluble drug that has a low dissolution rate and a more soluble coformer may facilitate dissolution improvement of the low-solubility API, even compared to its own pure amorphous form.

Coamorphous formulations using low-molecular-weight excipients can include non-pharmaceutically active, generally recognized as safe (GRAS) compounds. Alternatively, a second drug molecule can be used to provide a dual-action pharmaceutical formulation, such as coamorphous indomethacin/paracetamol,^18^ naproxen/cimetidine,^17^ and indomethacin/naproxen,^19^ which all show greater physical stability and enhanced dissolution rates as well as evidence of intermolecular interactions between components. Other drug–drug coamorphous solids, such as simvastatin/glipizide^20^ and ritonavir/indomethacin,^21^ show dissolution and stability improvements without any evidence of specific intermolecular interactions. However, in both of these cases, while the dissolution rate of one drug component was increased compared to its pure amorphous form, the second component did not see such an increase due to the lack of synchronous dissolution. Coamorphous formulations containing polymer excipients instead of small molecules are usually referred to as amorphous solid dispersions (ASDs), in which the stabilization of the amorphous API depends on its solubility in the carrier polymer.^22^ Below the miscibility limit, the drug is molecularly dispersed and stabilized against recrystallization by physical separation of the drug molecules between polymer chains.^22^ The polymer matrix usually raises the *T*_g_ of the ASD compared to the pure amorphous drug, inhibiting crystallization through a reduction of molecular motion to impart greater physical stability,^23^ and it has been shown that differences in the types of intermolecular interactions between a given polymer and drug affect both the ASD dissolution performance and the maximum drug-loading (DL) capacity.^9,23,24^ Rapid dissolution of drug from an ASD produces a supersaturated solution with an enhanced free drug concentration that can exceed even the amorphous API solubility in some systems; in these cases, the drug separates from the bulk aqueous phase via liquid–liquid phase separation to produce colloidal nanoparticles, which act as a reservoir to maintain the elevated drug concentration.^9,24,25^ This behavior is generally at lower drug loadings where drug and polymer are released congruently at the rate of polymer dissolution—the limit of this congruent release is referred to as the “LoC” of the ASD formulation. The method used to prepare ASDs with acidic polymers has been shown to have a strong impact on the degree of proton transfer between drug and polymer, particularly for combinations of drug and polymer that differ in p*K*_a_ by several log units, where the greater charge separation between drug and polymer causes greater aqueous solubility.^26^ ASDs of the malaria drug lumefantrine and poly­(acrylic acid) (PAA) produced by slurry conversion showed 70% protonation of the drug by the polymer in the resulting solid, whereas the same ASDs prepared by melt quenching showed only 20% protonation and had 6-fold lower apparent solubility in simulated gastric fluid.^26^ On the downside, ASDs are also quite often hygroscopic and, with water ingress, can cause a reduction in *T*_g_ (plasticization) that can lead to phase separation of the coamorphous solid and drug recrystallization.^27^ Furthermore, the DL of many ASDs is unable to exceed 25% w/w due to the limited miscibility of the drug with polymers, meaning that large volumes of polymer are required to produce the final dosage forms. Co-amorphous formulations with low-molecular-weight excipients are usually produced at a 1:1 molar ratio, by comparison.^28^ Designing an ASD formulation with optimized polymer selection and drug loading also relies solely on experimental information and cannot currently benefit from in silico screening protocols such as COSMOtherm,^29^ which aid in the development of low-molecular-weight multicomponent solids.^30^

While there are no reports in the literature of PROTAC coamorphous formulations using low-molecular-weight coformers, there are several recent examples of successfully applying ASD formulations to enhance the solubility of PROTAC compounds. Pöstges et al. first produced ASD formulations of an initially amorphous androgen-receptor PROTAC “ARCC-4” at 10% and 20% DL with hydroxypropyl methyl cellulose acetate succinate (HPMCAS) and Eudragit polymers by vacuum compression molding, with nonsink dissolution studies showing a pronounced supersaturation enhancement without drug precipitation.^31^ Hofmann et al. also demonstrated significant supersaturation enhancements for spray-dried ASDs of an initially amorphous cereblon (CRBN) PROTAC with Soluplus and Eudragit polymers at 10% DL, compared to the pure amorphous API.^32^ Mareczek et al. later demonstrated dissolution enhancements for spray-dried ASDs of both an initially semicrystalline PROTAC “ARV-110” and an initially amorphous PROTAC “SelDeg51” with poly­(vinyl alcohol) (PVA) at 30% DL, indicating that the ASD formulations using the crystalline API were physically stable for at least 4 weeks.^33^ Most recently, Zhang et al. studied ASDs of a CRBN PROTAC with HPMCAS, Eudragit and Soluplus prepared at 5, 10, and 20% DL by solvent evaporation, confirming the presence of drug-polymer hydrogen-bonding interactions using FTIR and showing that HPMCAS ASDs could be produced as high as 40% DL, although ASDs at higher drug loading showed poor dissolution performance.^34^ They also showed, however, that the limited dissolution enhancement of the high DL ASDs could be improved greatly by adding sodium dodecyl sulfate as a surfactant to produce a ternary ASD system.^34^ However, these studies did not show how the dissolution enhancement of a given PROTAC ASD varied with the ASD preparation method used and, with the exception of work by Mareczek et al., whether the selected preparation method could be applied successfully to more than one PROTAC compound. Furthermore, the previous studies have not investigated the slurry conversion method for producing ASDs, which was shown by Neusaenger et al. to produce lumefantrine–PAA ASDs with the highest degree of drug protonation and therefore the greatest aqueous dissolution enhancement compared to other methods.^26^ In this work, we compare the dissolution behavior of coamorphous formulations of a CRBN PROTAC “AZ1” using either small molecule or polymeric excipients and investigate the nature of noncovalent interactions between drug and excipient components. We also investigate the effect of polymer selection and DL and ASD preparation methods on the dissolution performance of AZ1 ASDs and establish that the results are general across four structurally distinct CRBN PROTACs.

## Experimental Section

### Materials and General Methods

PROTAC compounds “AZ1–4” were supplied by AstraZeneca and were prepared according to previously published methodology.^35^ AZ1 amorphous form A was prepared by neat milling of AZ1 powder as synthesized using a Retsch MM200 Mixer Mill for 20 min at 20 Hz in a stainless steel milling jar. Hydroxypropyl methyl cellulose acetate succinate (HPMCAS-LG–AQOAT) was supplied by Shin-Etsu Chemicals (Tokyo, Japan). Polyvinylpyrrolidone vinyl acetate (PVPVA–Kollidon VA64) was supplied by the BASF Corporation (Ludwigshafen, Germany). All other chemicals and solvents were available from commercial sources and used without further purification. Infrared spectra were recorded between 4000 and 550 cm^–1^ using a PerkinElmer 100 FT-IR spectrometer with a μATR attachment. Unless otherwise specified, powder X-ray diffraction (XRPD) patterns were collected at room temperature using a Bruker AXS D8 Advance GX003410 diffractometer with a Lynxeye Soller PSD detector, using Cu Kα radiation at a wavelength of 1.5406 Å and collecting from 2° ≤ 2θ ≤ 40°.

### COSMOconf and COSMOtherm Calculations

COSMOconf and COSMOtherm (version 23.0.0) were used to calculate excess enthalpy/mixing enthalpy of the interaction between drug and coformers, using up to ten of the lowest energy conformers generated by COSMOconf. Drugs and coformers were entered in SMILES notation to calculate the sigma profiles. The optimized geometries of the structures were obtained using TZVP-FINE parametrization to obtain the necessary COSMO files. Calculation of the interaction between drug and coformer components was performed using COSMO (conductor-like screening model) software and BP_TZVPD_FINE_19 parametrization. The polar and hydrogen-bond interaction energies between the components are quantified based on the surface screening charge densities, which result from a quantum chemical continuum solvation calculation using COSMO.^29,36^ The strength of interactions between the two components, as compared with that of the pure reactants, is estimated via the mixing enthalpy. Mixing enthalpy is a rough approximation of the free energy of interaction. The greater (more negative) the mixing enthalpy, the greater the hetero (drug–coformer) interactions, and the greater the probability of cocrystal formation.

### Mechanochemical Milling of PROTACs

PROTAC compounds AZ1–4 were amorphized and reduced to smaller, homogeneous particles for dissolution studies by adding PROTAC powder to a 5 mL agate grinding jar with one agate grinding ball (6.4 mm diameter) and grinding with a Retsch MM200 Mixer Mill at 20 Hz for 20 min. The resulting solids were characterized by XRPD, modulated differential scanning calorimetry (mDSC), thermogravimetric analysis (TGA), FTIR, and scanning electron microscopy (SEM).

### AZ1 Cocrystal Screening

AZ1 (5 mg) and coformers were mixed in an equimolar ratio in HPLC vials, and ethyl acetate (5 μL) was added before liquid-assisted grinding (LAG) for 1 h at 600 rpm with one stainless steel ball bearing in a Fritsch Pulverisette 7 planetary ball mill. The resulting solids were analyzed by XRPD using a Rigaku SmartLab diffractometer with Cu Kα radiation at a wavelength of 1.5406 Å and collecting from 3° ≤ 2θ ≤ 40°. Fully amorphous solids were analyzed further by mDSC.

### Preparation of AZ1 Coamorphous Salts

Salts of AZ1 with oxalic acid (OXA), m-nitrobenzoic acid (NBA), and α-ketoglutaric acid (KGA) were prepared by adding AZ1 and coformer (50 mg total mass) in an equimolar ratio to a 5 mL stainless steel grinding jar with one stainless steel grinding ball (6.4 mm diameter) and LAG with a Retsch MM200 Mixer Mill at 20 Hz for 20 min. The resulting solids were analyzed by XPRD, heat–cool–heat mDSC, TGA, and FTIR.

### Preparation of ASDs by Slurry Conversion

Powders of PROTAC compounds AZ1–4 and polymers were mixed in a vial at the intended DL (% w/w), and dichloromethane–ethanol (1:1 v/v) was added to the powder at a solid/liquid ratio of 1:4 w/w. Each batch contained approximately 50–60 mg of the PROTAC compound. The slurries were stirred magnetically for 5 min at 70 °C in a water bath and dried under vacuum at room temperature for 24 h. The solid products were added to a 5 mL agate grinding jar with one agate grinding ball (6.4 mm diameter) and ground with a Retsch MM200 Mixer Mill at 20 Hz for 20 min. The resulting solids were characterized by XRPD, mDSC, TGA, FTIR, and SEM.

### Preparation of ASDs by Mutual Solvent Evaporation

AZ1 ASDs using HPMCAS and PVPVA were generated by dissolving both polymer and PROTAC components in dichloromethane–ethanol (4:1 v/v) before evaporating the solvent in an oven at 70 °C overnight, producing ASDs at 10%, 20%, 30%, and 40% w/w DL. Powder masses and solvent volumes were used such that all ASDs contained the same mass of AZ1 and had the same surface area, varying in the total ASD mass.

### Thermal Analysis

DSC samples were prepared using Tzero standard pans and lids with pinholes and analyzed using a TA Instruments Q2000 differential scanning calorimeter. Single-ramp mDSC samples were first equilibrated at 25 °C, then heated to 200 °C using a modulated method with a scanning speed of 3 °C/min, an amplitude of ±1 °C, and a period of 60 s. Heat–cool–heat mDSC samples were heated from 20 °C to 200 °C, cooled to 25 °C, and reheated to 200 °C. The instrument was calibrated using an indium standard prior to analysis, with a melting point onset of 156.89 °C and a heat capacity of 33.971 J/g. TGA samples were analyzed using platinum pans and a TA Instruments Discovery thermogravimetric analyzer, heating from 25 to 200 °C at 20 °C/min.

### Scanning Electron Microscopy

SEM samples were prepared by adding solids to polycarbonate wafers and coating with 25 nm of platinum using a Cressington 328 ultra-high-resolution EM coating system. The images were obtained using a Carl Zeiss Sigma 300 VP FEG SEM microscope operated at 5 kV using an in-lens detector.

### Ultra Performance Liquid Chromatography (UPLC) Analysis

The concentrations of PROTAC compounds were determined using a Waters ARC UPLC -MC206 system with an ACQUITY UPLC BEH C18 column (130 Å, 1.7 μm, 2.1 mm × 50 mm, Waters Corporation, UK) and a UV detection wavelength of 300 nm. The mobile phase of acetonitrile/water varied in a gradient method from 95/5 to 5/95 v/v at a flow rate of 1 mL/min.

### PROTAC Solubility

The thermodynamic solubilities of amorphous PROTACs AZ1–4 in fasted state simulated intestinal fluid (FaSSIF) were determined by adding an excess of amorphous PROTAC to 1 mL of solvent and stirring at 1000 rpm for 24 h. The samples were then centrifuged for 30 min at 31,000 × *g,* and the supernatants were diluted appropriately to maintain absorbance readings within the UPLC standard curve. The concentration of PROTAC was determined by UPLC analysis, converting peak area values to concentrations via a calibration curve. Solid residues were taken from each slurry after 24 h and analyzed by XPRD to confirm that the PROTACs were still amorphous.

### nonsink Powder Dissolution Measurements

Dissolution experiments were performed in duplicate for each sample. The powders were sieved by using standard mesh sieves to remove particles larger than 150 μm. Vessels were charged with accurately weighed masses of the amorphous PROTAC and ASD solids before adding the correct volume (approximately 10 mL) of prewarmed FaSSIF at 37 °C such that all slurries were accurately at ten times the measured solubility limit of the amorphous PROTAC. Slurries were stirred at 400 rpm for 2 h. Aliquots of the slurries were removed at each time point, centrifuged for 30 min at 31,000 × *g*, and the neat supernatant was analyzed to maintain absorbance readings within the UPLC standard curve. The concentrations of PROTAC were determined by UPLC analysis, converting peak area values to concentrations via a calibration curve. The pH of each dissolution slurry was recorded at the end of the experiment to confirm that it had not varied outside of the specification of the buffer. Solid residues were taken from each slurry at the end of the dissolution experiment and analyzed by XRPD to confirm that the samples did not recrystallize during the experiment.

### ASD Conditioning

Samples of ASDs found to provide a dissolution advantage over the pure drug substance were conditioned in a desiccator at 40 °C and 75% relative humidity (RH) using a saturated NaCl solution,^37^ and the resulting solids were analyzed by XRPD, mDSC, and TGA.

## Results and Discussion

Compound AZ1 is a PROTAC consisting of an imide-based ligand that recruits CRBN, and an ER ligand adjoined via a piperazine-/piperidine-based linker moiety (Scheme 1). We have previously shown that AZ1 is able to crystallize in an anhydrous form and two solvate forms; however, it is very difficult to grow these crystals, and the vast majority of crystallization attempts yielded amorphous material.^35,38^ Amorphous solids prepared by either milling an AZ1 anhydrous phase or desolvating a solvate crystal also exhibit very different dissolution behavior. In this work, we will consider only the amorphous “form A” produced by milling the anhydrous solid, a form characterized by a broad halo in the X-ray powder diffractogram and a high *T*_g_ of 160 °C. This amorphous solid has an equilibrium solubility of 48.4 ± 2.6 μg/mL in fasted state simulated intestinal fluid (FaSSIF) at 37 °C. Coamorphous formulations of this compound with low molecular weight and polymeric excipients are compared herein. PROTAC compounds AZ2, AZ3, and AZ4 consist of the same ligand moieties as AZ1 but with different linker groups (Scheme 2), and are all amorphous in their as-synthesized form^35^ with even higher *T*_g_ values around 170–174 °C. The equilibrium solubilities of AZ2, AZ3, and AZ4 in FaSSIF at 37 °C are 28.1 ± 5.2, 34.5 ± 7.7, and 17.3 ± 1.6 μg/mL, respectively. Since these compounds are available in much lower quantities, only the most effective coamorphous formulation strategy for dissolution enhancement of AZ1 was taken forward to study these PROTACs.

**Scheme 1:**
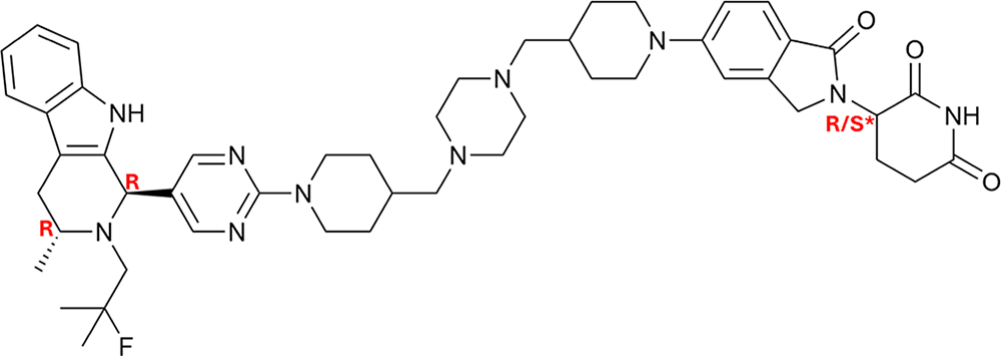
CRBN PROTAC Compound AZ1^a^

a The indole-containing moiety shown on the left constitutes the estrogen receptor (ER) ligand or “ER-warhead.” The imide-based moiety shown on the right constitutes the “CRBN-ligand.” The two are joined chemically via a piperazine/piperidine-based linker. The chiral center marked by an asterisk * is present as an equimolar mixture of R and S configurations to give a 1:1 mixture of RRR and RRS diastereomers.

**Scheme 2:**
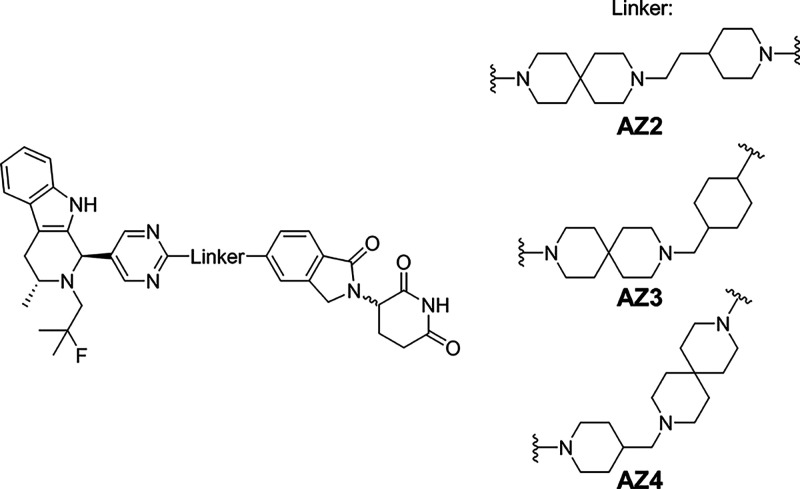
PROTAC Compounds AZ2, AZ3, and AZ4^a^

a Molecules differ from each other and AZ1 only by the structure of the linker moiety.

A list of 48 small molecules was chosen for a cocrystal screen with AZ1 based on a combination of in silico predictions and consideration of potential supramolecular synthons between components. Molecules previously shown to cocrystallize with lenalidomide, a drug compound that structurally resembles the CRBN-ligand moiety of AZ1, were also included in the cocrystal screen with AZ1.^39–44^ The solids produced by LAG of each coformer with AZ1 in the presence of ethyl acetate were analyzed by XRPD, which showed that all samples containing crystalline material of any kind contained only separate phases of the original components, either as a convolution of the powder patterns for the two starting materials or as the crystalline pattern of one component convoluted with a broad amorphous halo from the second component. All samples that produced a completely amorphous powder pattern were analyzed further by mDSC to determine whether the amorphous solid consisted of separate, physically mixed amorphous components or a single coamorphous phase. The presence of only one glass transition temperature (*T*_g_) at a lower temperature than for pure AZ1 indicates that the sample is a single coamorphous phase rather than a physical mixture of components. Nine of the forty-eight LAG experiments produced amorphous solids with a single *T*_g_ varying in breadth between the samples, indicating different degrees of heterogeneity depending on the coformer (ESI Figures S1 and S2).

Three of the nine coamorphous solids with KGA, NBA, and OXA as coformers were chosen for scaled-up synthesis and nonsink dissolution comparison against pure AZ1 based on a combination of three criteria. Since the *T*_g_ values of AZ1 and other PROTACs are often high due to low molecular mobility and high physical stability of the amorphous phase, and may be correlated to their poor dissolution performance, coamorphous solids with the lowest *T*_g_ were favored. The physical stability of the solids after three months was assessed by XRPD (ESI Figure S3), with samples that had recrystallized into separate components over this duration deemed physically unstable and unsuitable for further study. Finally, the coamorphous solids were ranked by the aqueous solubility of the pure coformer, based on the hypothesis that if strong intermolecular bonds can form between the coformer and PROTAC and enable synchronous dissolution, a more soluble coformer may produce a coamorphous solid with a greater dissolution advantage over the pure API. It should be noted that a more rapidly dissolving coformer is less likely to destabilize the amorphous phase and lead to the recrystallization of poorly crystallizable PROTACs, as it would for smaller conventional drugs.^22^ AZ1 has a calculated p*K*_*a*_ of approximately 10, while NBA, the least acidic of the three coformers, has a p*K*_a_ of 3.5; therefore, these three solids may exist in the form of coamorphous salts, assuming that the “Δp*K*_a_ rule” used to predict the formation of cocrystalline materials also holds true for amorphous solids. This rule states that where there is a difference of more than 2–3 log units between the p*K*_a_ of the acid and protonated base components, salt formation is likely in the resulting two-component solid.^45–47^ While other AZ1 coamorphous solids, such as those of saccharin and salicylic acid, had a significantly lower *T*_g_ than AZ1-OXA and AZ1-KGA, the coformers recrystallized from the solids within days. This is likely because the lower *T*_g_ indicates greater molecular mobility, and even if stabilized by drug–coformer intermolecular interactions, these amorphous glasses appear less kinetically stable overall.

FTIR analysis (Figure 1) shows that despite the presence of both components in an equimolar ratio in all three coamorphous solids, the spectra are all dominated by signals from AZ1 due to its much greater number of atoms. There are some small differences in the spectra for the AZ1-OXA and AZ1-KGA solids compared to amorphous AZ1, with relative growth in intensity, sharpening of the carboxylic acid ν_(C=O)_ band at 1708 cm^–1^ compared to the band at 1683 cm^–1^, and a change in the relative strengths of bands at 1012 and 989 cm^–1^ in the alkene ν_(C=C)_ bending region. However, the spectrum of AZ1-NBA contains many more features that are shifted relative to the pure components. The presence of a new carboxylic acid ν_(C=__O)_ band at 1732 cm^–1^, shifts of 3–20 cm^–1^ in both directions for the nitro ν_(N–O)_ stretches and shifts of 6–14 cm^–1^ in both directions for the alkene ν_(C=C)_ bending modes suggest that the molecules may be engaged in hydrogen-bonding interactions via both the carboxylic acid and nitro moieties of NBA, most likely with the carbonyl and imide moieties in AZ1. There is no strong evidence to suggest that intermolecular interactions form between AZ1 and OXA or KGA, with these two coamorphous solids having very similar spectra to each other.

**Figure 1:**
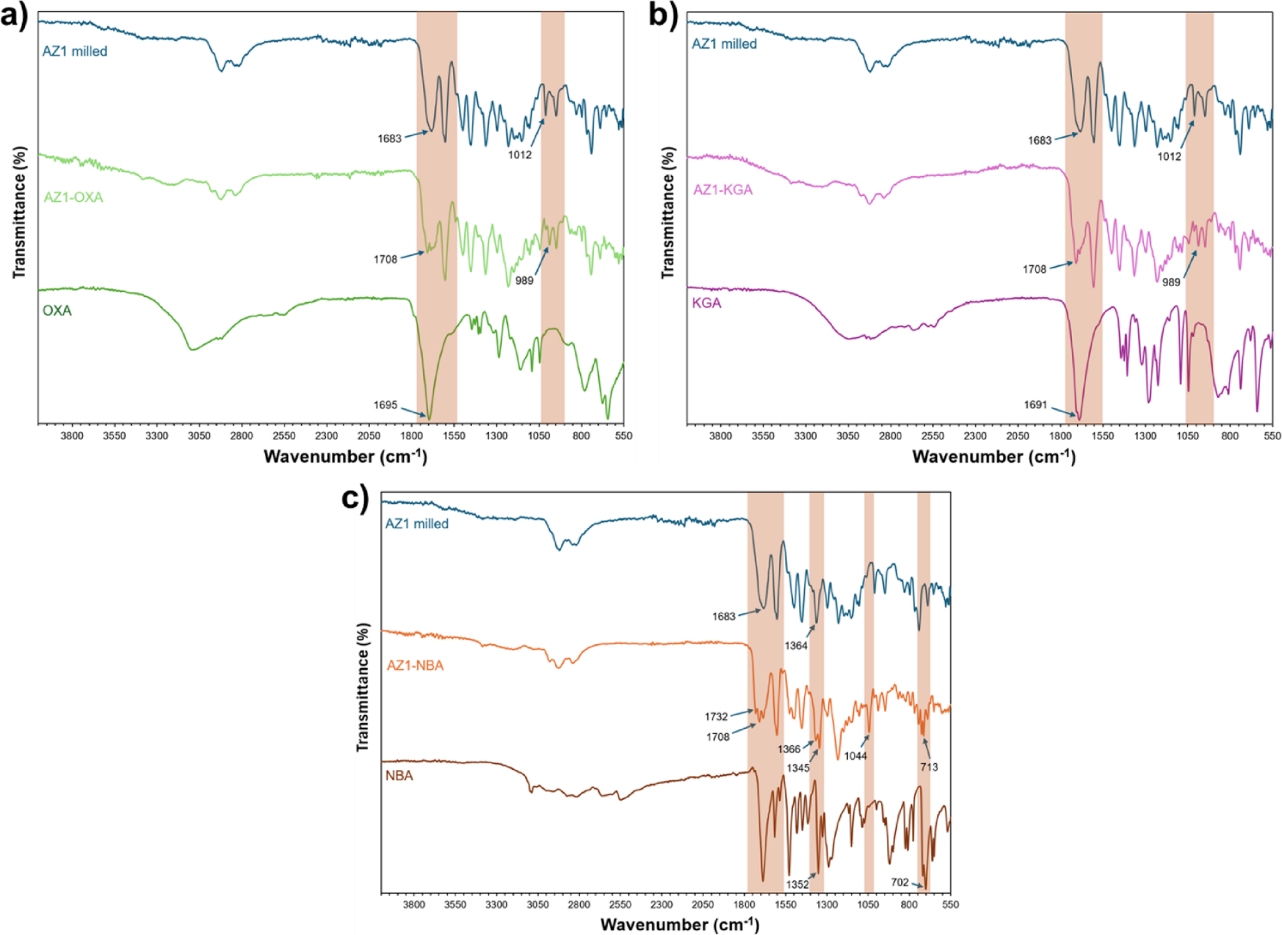
FTIR spectra of AZ1 coamorphous solids with (a) OXA, (b) KGA, and (c) NBA compared to the pure components.

MDSC (Figure 2a) shows that AZ1-NBA, AZ1-KGA, and AZ1-OXA have *T*_g_ values of 86 °C, 136 °C, and 157 °C, respectively, with AZ1-NBA showing a relatively broad glass transition event. While AZ1-OXA does not have a significantly lower *T*_g_ than pure AZ1, the solubility of the coformer and the physical stability of the coamorphous solid over 3 months made it a suitable candidate for dissolution studies. AZ1-NBA, on the other hand, shows a very significant difference in *T*_g_ compared with the pure API. TGA (ESI Figure S4) shows that all three solids are dry with no significant mass loss up to 120 °C, followed by gradual mass loss totaling approximately 5% upon heating from 120 °C to 200 °C. This is likely due to samples degrading as they exceed *T*_g_.

**Figure 2:**
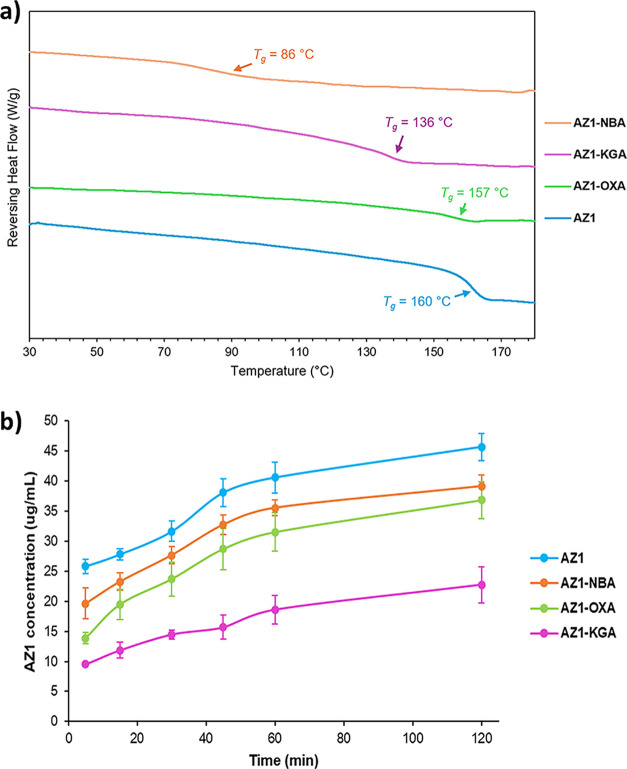
(a) Modulated DSC thermograms for AZ1 and its coamorphous salts with OXA, NBA, and KGA. The *T*_g_ of AZ1-KGA and AZ1-NBA are significantly reduced compared to the pure drug. (b) nonsink dissolution profiles of amorphous AZ1 and coamorphous salts with NBA, OXA, and KGA over 2 h in fasted state simulated intestinal fluid (FaSSIF) at 37 °C, using a 10-fold nonsink condition compared to the amorphous solubility. Dissolution experiments were run in duplicate, with average concentrations plotted and error bars showing the range of concentrations measured at each time point.

Nonsink dissolution profiles for amorphous AZ1 and the three coamorphous salts over 2 h in FaSSIF at 37 °C are shown in Figure 2b, allowing for discrimination between the dissolution profiles of the pure API and the coamorphous formulations. AZ1 powder was milled via the same method used to produce the coamorphous phases, except that no ethyl acetate solvent was used to reduce and homogenize the particle size, and all solid samples were sieved to remove particles larger than 150 μm. All three coamorphous salts have a similar dissolution profile shape to amorphous AZ1, but at lower apparent solubilities over the 2 h range, with AZ1-KGA exhibiting the lowest solubility at approximately 40% of the concentration of AZ1 in solution at every time step. This may be partly due to their larger particle sizes of roughly 10–60 μm as observed by SEM (ESI Figure S5), being more comparable to the ripened AZ1 particles after 24 h of slurry rather than the smaller particles initially produced by the milling process (ESI Figure S6), and also because in the cases of AZ1-OXA and AZ1-KGA there are no strong drug–coformer interactions to enable synchronous dissolution despite the high aqueous solubilities of the coformers. AZ1-NBA has the best dissolution performance of the three coamorphous salts, which may be related to the presence of stronger intermolecular interactions between components as observed by FTIR analysis. However, even in this example, the interactions are too weak to confer a dissolution advantage compared to the pure amorphous API. Despite the relative ease of production, coamorphous formulations with small molecule coformers do not appear to be an effective strategy for PROTAC dissolution enhancement.

### ASDs of AZ1

Five commercial polymers commonly used in pharmaceutical products^48^ were selected for preliminary screening of ASD formation with AZ1: hydroxypropyl methyl cellulose (HPMC), HPMCAS, PAA, poly­(vinylpyrrolidone) (PVP), and poly­(vinylpyrrolidone)/vinyl acetate (PVPVA). Small-scale ASDs were produced directly within DSC and TGA pans by solvent evaporation of drug–polymer solutions to achieve a drug loading (DL) of 10–40% w/w prior to thermal analysis. HPMCAS and PVPVA reliably formed ASDs with clearly measurable *T*_g_ values, while the others produced lower quality thermal data, often with undetectable *T*_g_ events. HPMCAS and PVPVA were therefore taken forward to investigate the effects of polymer selection on the drug loading and dissolution advantages that can be achieved by ASD formulations of AZ1. HPMCAS is substituted with acetyl and succinoyl groups, resulting in an approximate p*K*_a_ of 5, while PVPVA is much less acidic with a p*K*_a_ similar to protonated AZ1 at around 9.5, providing the opportunity to study the role of polymer acidity in ASD formation and performance. The slurry conversion and solvent evaporation methods of preparing these ASDs were also compared, since the ASD preparation method has been shown to affect the degree of proton transfer between drug and polymer and therefore impact aqueous dissolution.^26^

### Slurry Conversion ASDs

All ASDs of both polymers from 10% to 40% w/w DL prepared by the slurry conversion method showed a single *T*_g_ and an amorphous X-ray powder pattern (ESI Figure S7), indicating the formation of a single coamorphous phase. TGA (ESI Figures S8 and S9) shows that the HPMCAS ASDs contain 0.5% w/w residual solvent on average, even after drying. PVPVA ASDs contain even more residual solvent, with 1–3% weight loss observed between 20 and 80 °C. Both HPMCAS and PVPVA ASDs show a roughly linear positive correlation between DL and *T*_g_ observed by mDSC (Figure 3), trending upward toward the *T*_g_ of pure AZ1. While the measured *T*_g_ values of the PVPVA ASDs match the values calculated using the Fox relation (a simplified form of the Gordon–Taylor equation^49^) to within 1–2 °C, the *T*_g_ values measured for the HPMCAS ASDs are all 4 °C higher than the predicted values from 10% to 30% DL before matching the prediction exactly at 40% DL (ESI Table S1). The Fox equation assumes ideal mixing with no excess enthalpy or entropy. A positive deviation in *T*_g_ values, such as this, is likely indicative of stronger intermolecular drug-polymer interactions in the HPMCAS ASDs up to 30% DL, while the PVPVA ASDs behave more like ideal mixtures across the 10–40% DL range. This may arise from stronger interactions between the basic API and the more acidic HPMCAS polymer compared to PVPVA. While these strong interactions may enhance dissolution performance compared to the PVPVA ASDs, work by the Taylor group has shown that they can also be correlated with a lower LoC and therefore greater potential pill burden.^9,49^ The FTIR spectra (ESI Figures S10 and S11) of all ASDs are dominated by the polymer signals at low DL with very little change as the DL increases, except for the growth of the AZ1 carboxylic acid ν_(C=O)_ bands at 1613 and 1683 cm^–1^, which are not shifted relative to the pure drug substance. This suggests that there is no hydrogen-bonding interaction between components, although it does not rule out dispersive forces such as aliphatic stacking interactions, which have been shown in previous work to dominate the lattice energy of AZ1 crystal forms^38^ and could be present between drug and polymer chains. While X-ray photoelectron spectroscopy (XPS) was used by Neusaenger et al. to determine the degree of salt formation in amorphous lumefantrine-PAA dispersions prepared by slurry conversion,^26^ the presence of ten distinct nitrogen atoms in AZ1 makes it unfeasible to measure the degree of drug protonation by the same method, and there is no spectral evidence for drug-polymer proton transfer.

**Figure 3:**
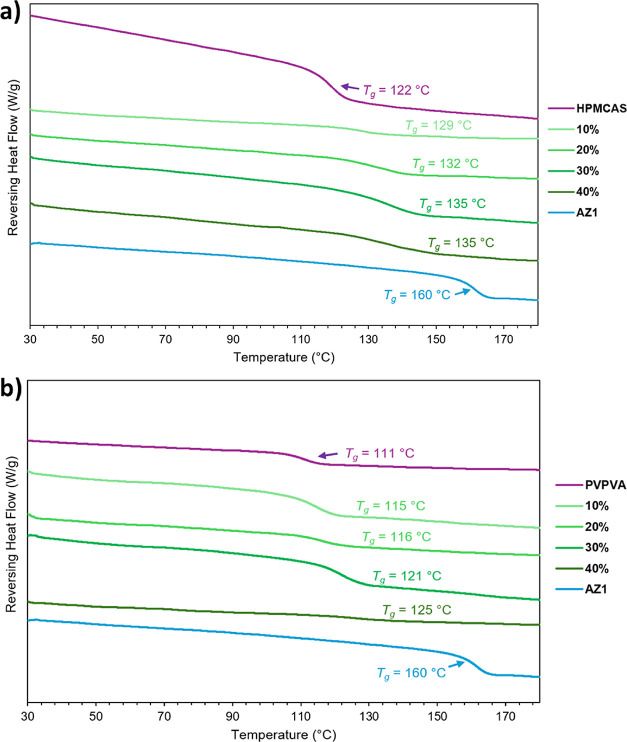
Modulated DSC thermograms for AZ1 and its ASDs from 10% to 40% DL with (a) HPMCAS and (b) PVPVA, prepared by slurry conversion. A trend toward higher *T*_g_ is observed as DL increases.

Milling the ASDs to homogenize and reduce the particle sizes prior to dissolution studies produced smooth particles with a roughly flat-spherical or plate-like shape. Longer and/or faster milling did not reduce the particle size significantly further. While SEM (Figure 4) shows that milling the pure AZ1 powder produced particles ranging from approximately 1–5 μm, the ASD particles were larger at 10–50 μm.

**Figure 4:**
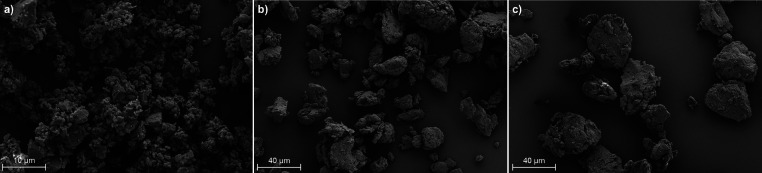
SEM images of (a) milled AZ1, (b) AZ1 HPMCAS ASD (10% DL), and (c) AZ1 PVPVA ASD (10% DL).

Nonsink dissolution profiles for amorphous AZ1 and the HPMCAS and PVPVA ASDs over 2 h in FaSSIF at 37 °C are shown in Figure 5. The milled amorphous AZ1 dissolves at a higher apparent solubility of approximately 65 μg/mL compared to the equilibrium solubility at 48 μg/mL measured over 24 h in FaSSIF, likely because the micronized particles have a higher surface area and may dissolve quickly before reprecipitating as larger particles over 24 h. This Ostwald ripening of amorphous particles has been exemplified with felodipine by Olsson and co-workers,^50^ and was confirmed in this case by SEM analysis of the particles before and after 24 h of slurry, showing that the particles had grown from an initial 1–5 up to 25–50 μm (ESI Figure S6). All the HPMCAS ASDs show further increases in apparent solubility compared to AZ1, with the ASDs at 10 and 20% DL showing almost double the solubility in the first 5 min and a sustained dissolution increase over the full 2 h duration, maintaining at least 10% higher solubility after 2 h. However, HPMCAS ASDs at 30 and 40% DL follow the same profile as pure AZ1, indicating that the limit of congruency (LoC) for these ASDs is between 20% and 30%, but the presence of some polymer in solution may be the cause of the slightly enhanced dissolution compared to the pure drug. This LoC is reasonably high, given that the deviations in *T*_g_ compared to predicted values would suggest strong interactions between components.^9,51^ The dissolution profiles of all HPMCAS ASD samples are also relatively consistent between repeats. This shows that despite the particles being roughly an order of magnitude larger than pure AZ1, the HPMCAS ASD formulations at 10 and 20% DL are capable of dissolving AZ1 to a higher concentration and more quickly than the pure drug powder alone. By contrast, none of the PVPVA ASDs showed a different shape of dissolution profile to pure AZ1, although ASDs at 20–40% DL appear to have slightly higher apparent solubility over the first 45 min of dissolution compared to pure AZ1. However, there was also much greater variance in the profiles between repeated measurements of the same PVPVA samples, suggesting sample inhomogeneity despite the milling process. These results indicate PROTAC ASD formulations produced by the slurry conversion method can have a significant dissolution enhancement when using the correct polymeric excipient—in this case, HPMCAS appears to be a much more effective excipient than PVPVA, likely due to its greater acidity compared to the drug substance. This mirrors findings by the Taylor group that HPMCAS ASDs generally display higher drug release compared to PVPVA dispersions,^52^ and that the formation of drug-rich nanodroplets via liquid–liquid phase separation is observed for high-releasing ASD formulations when the drug concentration exceeded the amorphous solubility.^52–56^ It is proposed that HPMCAS is better at stabilizing these nanodroplets compared to PVPVA,^51^ creating a drug reservoir from which the high drug supersaturation can be maintained.^53–56^

**Figure 5:**
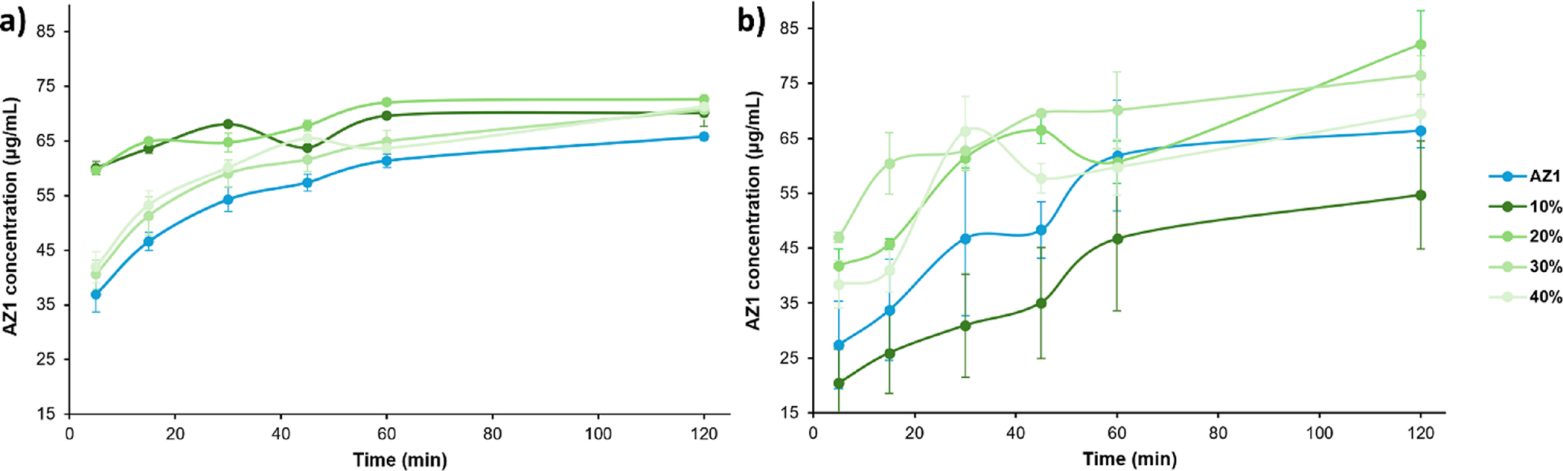
Nonsink dissolution profiles of amorphous AZ1 and (a) HPMCAS ASDs or (b) PVPVA ASDs of AZ1 prepared from 10 % to 40% DL by slurry conversion, over 2 h in FaSSIF at 37 °C and using a 10-fold nonsink condition compared to the amorphous solubility. Dissolution experiments were run in duplicate, with average concentrations plotted. Error bars indicate the range of concentrations measured at each time point.

### Solvent Evaporation ASDs

ASDs were also prepared by evaporation of a volatile solvent from a solution of AZ1 and polymer, producing thin films that contained no traces of crystalline material by XRPD analysis (ESI Figure S12). The FTIR spectra (ESI Figures S13 and S14) for ASDs of both polymers also closely matched the equivalent slurry conversion ASDs, showing no evidence of intermolecular interactions. The measured *T*_g_ values for the HPMCAS ASDs (Figure 6) were 4–7 °C higher than the calculated values, similar to those of the ASDs prepared by slurry conversion. The measured *T*_g_ values for the PVPVA ASDs ranged from 3 °C lower than the predicted value at 10% DL up to 8 °C higher than the predicted value at 40% DL, unlike those prepared by slurry conversion, which all matched the predicted values, suggesting that there are much stronger drug-polymer interactions present in the ASDs produced by this method. TGA analysis shows that HPMCAS ASDs contain slightly more residual solvent than when prepared by slurry conversion, with an average 1% weight loss up to 60 °C, and PVPVA ASDs contain slightly less, with 0.6% weight loss on average (ESI Figures S15 and S16). This is likely because the thin films produced were still slightly plasticized with solvent even after holding at 70 °C in an oven overnight.

**Figure 6:**
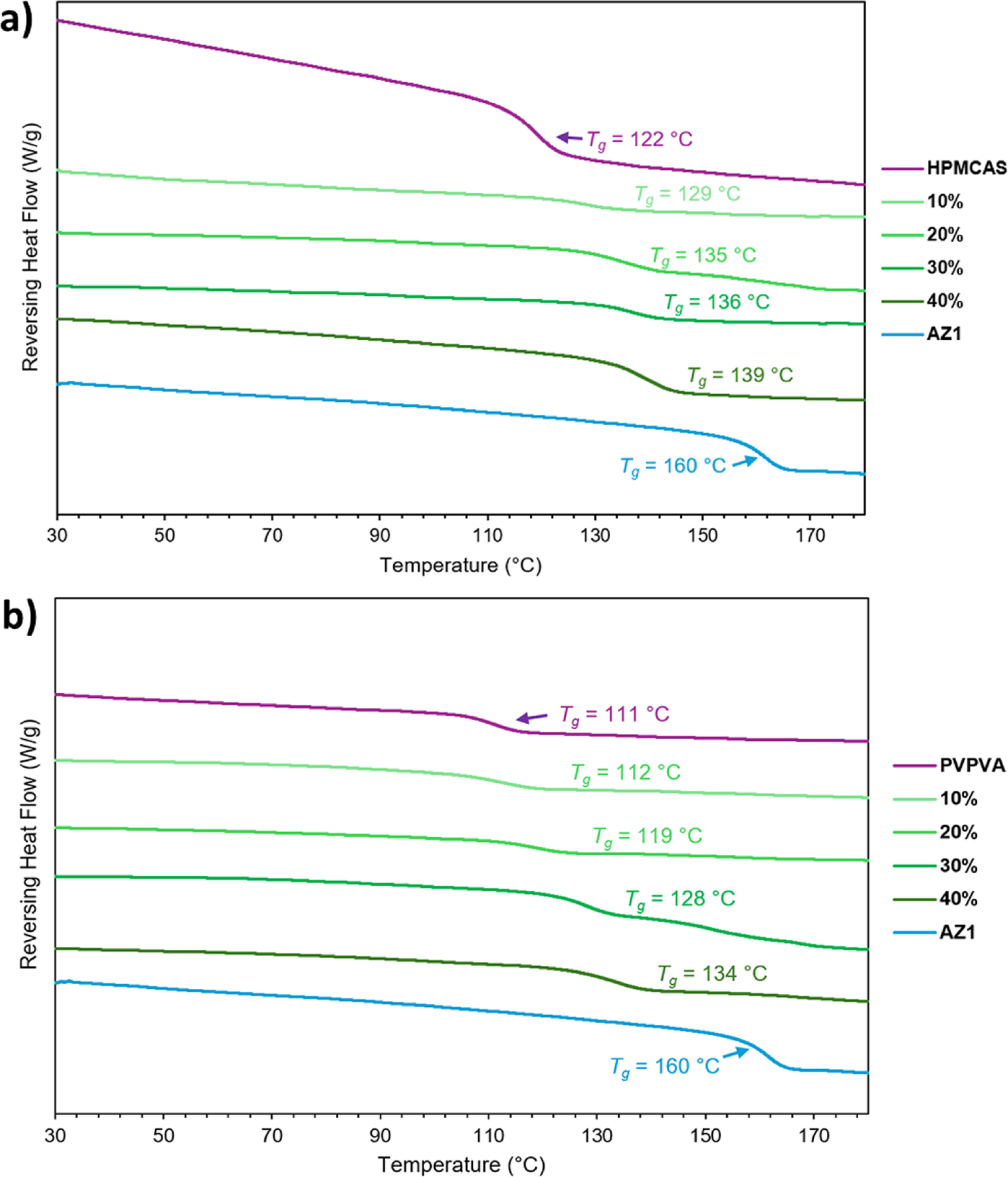
Modulated DSC thermograms for AZ1 and its ASDs from 10% to 40% DL with (a) HPMCAS and (b) PVPVA, prepared by solvent evaporation. A trend toward higher *T*_g_ is observed as DL increases.

Solvent evaporation ASDs were prepared such that the surface area of the ASDs produced with a given polymer would be constant, as well as containing a constant mass of AZ1, such that each dissolution experiment would contain a concentration of AZ1 at ten times the solubility limit measured for amorphous AZ1 in FaSSIF (10-fold nonsink condition). However, since pure AZ1 could not be manufactured as a thin film by the same method, the dissolution profiles measured for these ASDs are presented in comparison to that of AZ1 as a powder and are therefore not directly comparable, unlike for the slurry conversion ASDs.

Nonsink dissolution profiles for amorphous AZ1 and the HPMCAS and PVPVA ASDs prepared by solvent evaporation over 2 h in FaSSIF at 37 °C are shown in Figure 7. Only the 10% ASDs of both polymers show a consistent increase in apparent solubility over pure AZ1, noting again that the ASDs were prepared as thin films, whereas AZ1 was a free-flowing powder. ASDs prepared at 20% DL or higher with either polymer show no apparent solubility increase or have even a lower solubility than pure AZ1. While all PVPVA ASDs follow the same type of dissolution profile as AZ1, the 10% HPMCAS ASD demonstrates a spring in solubility in the first 15 min compared to the other samples, reaching approximately 33% higher than pure AZ1 before coming back down to follow the same profile as the pure drug. This is a considerably smaller dissolution improvement over pure AZ1 compared to the same ASD produced by slurry conversion, and it appears that the LoC for ASDs prepared by this method is also lower, at less than 20%. Any intermolecular interactions between AZ1 and PVPVA that were implied from the deviation of measured *T*_g_ values from the predicted values do not appear to have enhanced drug dissolution.

**Figure 7:**
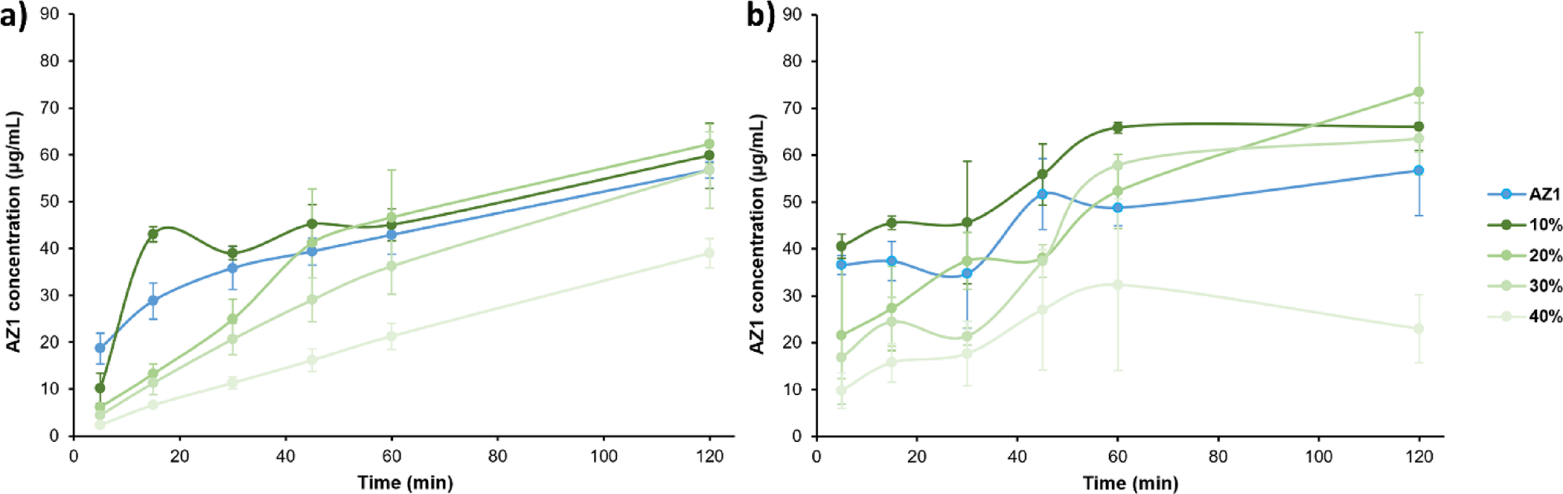
Nonsink dissolution profiles of amorphous AZ1 and (a) HPMCAS ASDs or (b) PVPVA ASDs of AZ1 prepared from 10 % to 40% DL by solvent evaporation, over 2 h in FaSSIF at 37 °C, and using a 10-fold nonsink condition compared to the amorphous solubility. Dissolution experiments were run in duplicate, with average concentrations plotted and error bars showing the range of concentrations measured at each time point.

Regardless of the preparation method, the HPMCAS shows significantly more promise than PVPVA for producing PROTAC ASD formulations with a dissolution advantage over the pure drug. This is likely because of stronger drug-polymer intermolecular interactions evidenced by a positive deviation in *T*_g_ compared to predicted values, and possibly also because the greater difference in p*K*_a_ between the two components facilitates a greater extent of proton transfer, producing a more strongly charge-separated solid which boosts aqueous solubility. It also appears that this can be achieved to a greater extent via the slurry conversion method rather than by mutual solvent evaporation, suggesting that the former method may facilitate greater separation of the drug within the polymer matrix or greater proton transfer between components.

### Applicability to Other PROTAC Compounds

The generality of the dissolution advantage provided by HPMCAS ASDs of a PROTAC produced via the slurry conversion method was tested by applying the same ASD formulation protocol to three other PROTAC compounds, AZ2–4 (Scheme 2). These compounds have the same ligand moieties but vary in the linker moiety and are all amorphous without milling, unlike AZ1. ASDs were prepared only as high as 30% DL since the highest LoC for AZ1 ASDs had been observed between 20% and 30%, and due to the limited available material for these additional PROTAC compounds. For the same reason, a comparison between polymers and/or ASD preparation methods was not possible for AZ2–4.

As for ASDs of AZ1, XRPD (ESI Figure S17) shows that all of the samples are amorphous. FTIR analysis of AZ2 ASDs (ESI Figure S18) shows downward shifts in the carboxylic acid ν_(C=O)_ band from 1730 to 1724 cm^–1^ for the 10% ASD, shifting incrementally upward with increasing DL back to 1730 cm^–1^ in the 30% ASD. The 10% ASD also has a narrower carboxylic acid ν_(C=O)_ band at 1705 cm^–1^. This seems to indicate the most/strongest hydrogen-bonding between AZ2 and HPMCAS at lower DL, decreasing toward higher DL. FTIR analysis of the AZ3 and AZ4 ASDs (ESI Figures S19 and S20) shows much less difference between the ASDs and pure components, with no discernible differences in the carbonyl region, suggesting an absence of hydrogen-bonding interactions between drug and polymer. TGA (ESI Figures S21–S23) shows that the AZ2 and AZ3 ASDs contain ∼0.3% residual solvent on average, with broad mass loss between 20 °C and 80 °C, with gradual mass loss totaling ∼1.5% over the remaining temperature range above 80 °C. AZ4 ASDs appeared to be dry below 80 °C but showed a steady mass loss between 120 °C and 200 °C of as much as 3%. DSC analysis (Figure 8) shows that for all three PROTACs, the ASDs increase in *T*_g_ with increasing DL, trending toward the respective pure PROTAC *T*_g_ values. Steeper trends in *T*_g_ with DL are observed in these samples compared to those of AZ1, since all three PROTACs have a higher *T*_g_ than AZ1 by 10–15 °C. The measured *T*_g_ values of all three sets of ASDs also show more significant positive deviations from the calculated *T*_g_ values, ranging from 5 °C to 14 °C higher than the predicted values. This suggests that there are stronger drug-polymer interactions in all three sets of ASDs compared with those of AZ1.

**Figure 8:**
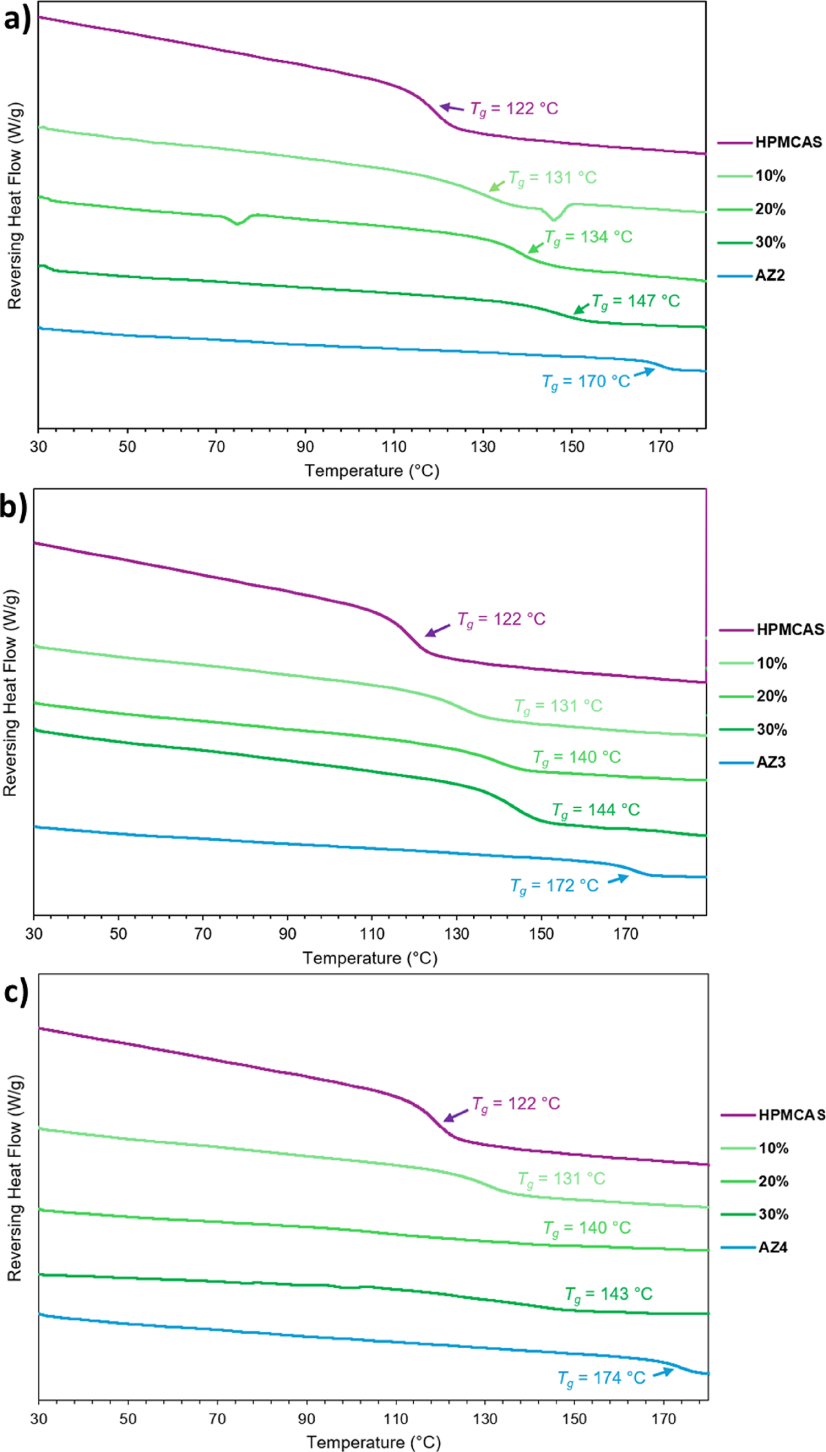
DSC thermograms of HPMCAS ASDs of (a) AZ2, (b) AZ3, and (c) AZ4 prepared by slurry conversion.

SEM (Figure 9) shows that milled AZ2 and AZ3 particles are approximately 1–5 μm, while milled AZ4 particles are 10–30 μm in size and appear to clump together more strongly. This is unlikely to be caused by a greater quantity of water adsorbed into the pure AZ4 drug substance since TGA shows that AZ4 contains less water than either AZ2 or AZ3 (ESI Figures S21–S23). ASD particles for all three PROTACs range from approximately 20 to 100 μm.

**Figure 9:**
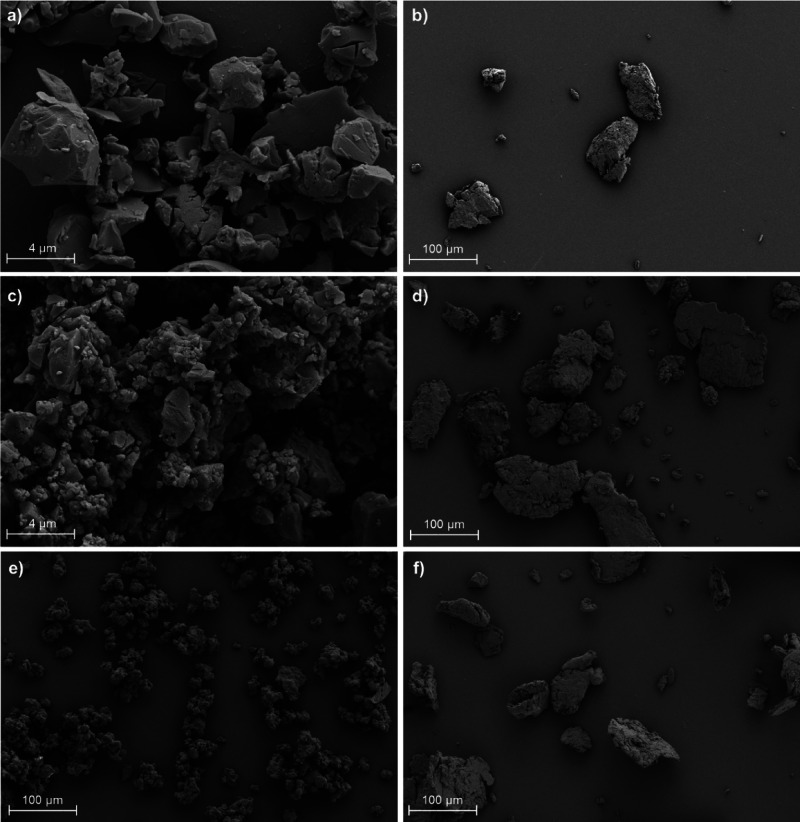
SEM images of (a) milled AZ2, (b) AZ2 HPMCAS ASD, (c) milled AZ3, (d) AZ3 HPMCAS ASD, (e) milled AZ4, and (f) AZ4 HPMCAS ASD. All ASDs are at 10% DL.

Nonsink dissolution profiles for amorphous AZ2, AZ3, and AZ4 and their ASDs over 2 h in FaSSIF at 37 °C are shown in Figure 10. All ASDs from 10% to 30% DL of both AZ2 and AZ3 show an increase in apparent solubility to varying extents, while only the 10% ASD of AZ4 shows such an increase, with 20% and 30% ASDs falling considerably lower in apparent solubility. This suggests that the LoC values of AZ2 and AZ3 ASDs may be even higher than 30%, with no limit detected in this study—these are far higher than expected given the considerable deviations in *T*_g_ from the predicted values. All three ASDs of AZ2 consistently show an approximate 50% boost in apparent solubility after 15 min and sustain a higher dissolution profile by no less than 15% across the full 2 h duration, with a significant spring in the first 30 min compared to pure AZ2. Interestingly, the highest DL sample at 30% shows the greatest spike in AZ2 concentration at 5 min, consistently between repeats. A similarly unexpected trend is observed for ASDs of AZ3, where increasing DL leads to greater overall apparent solubility, although with different profiles in each case. The 10% ASD exhibits an initial spring boosting to four times the drug concentration produced by pure AZ2, before falling back down to roughly the same profile as the pure drug after 1 h. The 20% ASD also starts with a significantly higher concentration after only 5 min, but rather than springing up and falling back down, the concentration increases to four times the apparent solubility of AZ2 and is sustained for the remainder of the dissolution experiment. The 30% ASD was by far the most soluble overall, measuring at over 17 times the apparent solubility of AZ2 after the first 5 min, but it has a flat dissolution profile compared to the other ASDs. The variation in the dissolution profiles for repeats of the AZ3 ASDs is also much greater, and the concentrations measured are much higher overall, since AZ3 is the most soluble of the PROTAC compounds studied. Meanwhile, ASDs of AZ4 all follow a similar dissolution profile to each other, with only the 10% ASD providing a spring in apparent solubility to approximately double the concentration of AZ4 compared to the pure drug substance after 15–30 min and sustaining no less than a 25% solubility boost compared to the pure drug over the 2-h range. ASDs at 20 and 30% consistently provide a 2–3 times lower concentration of AZ4 compared to the pure API across the dissolution experiment. The LoC for this system is clearly between 10% and 20% DL. The relatively poorer dissolution performance of AZ4 ASDs compared to the pure API contradicts the relatively lower difference in particle size between the pure and formulated samples, suggesting that it is more likely a result of weaker drug–polymer interactions. Alternatively, if drug-rich nanodroplets are formed by these ASD formulations, it may be that the HPMCAS polymer is less stabilizing for the AZ4 nanodroplets than for AZ2 or AZ3.

**Figure 10:**
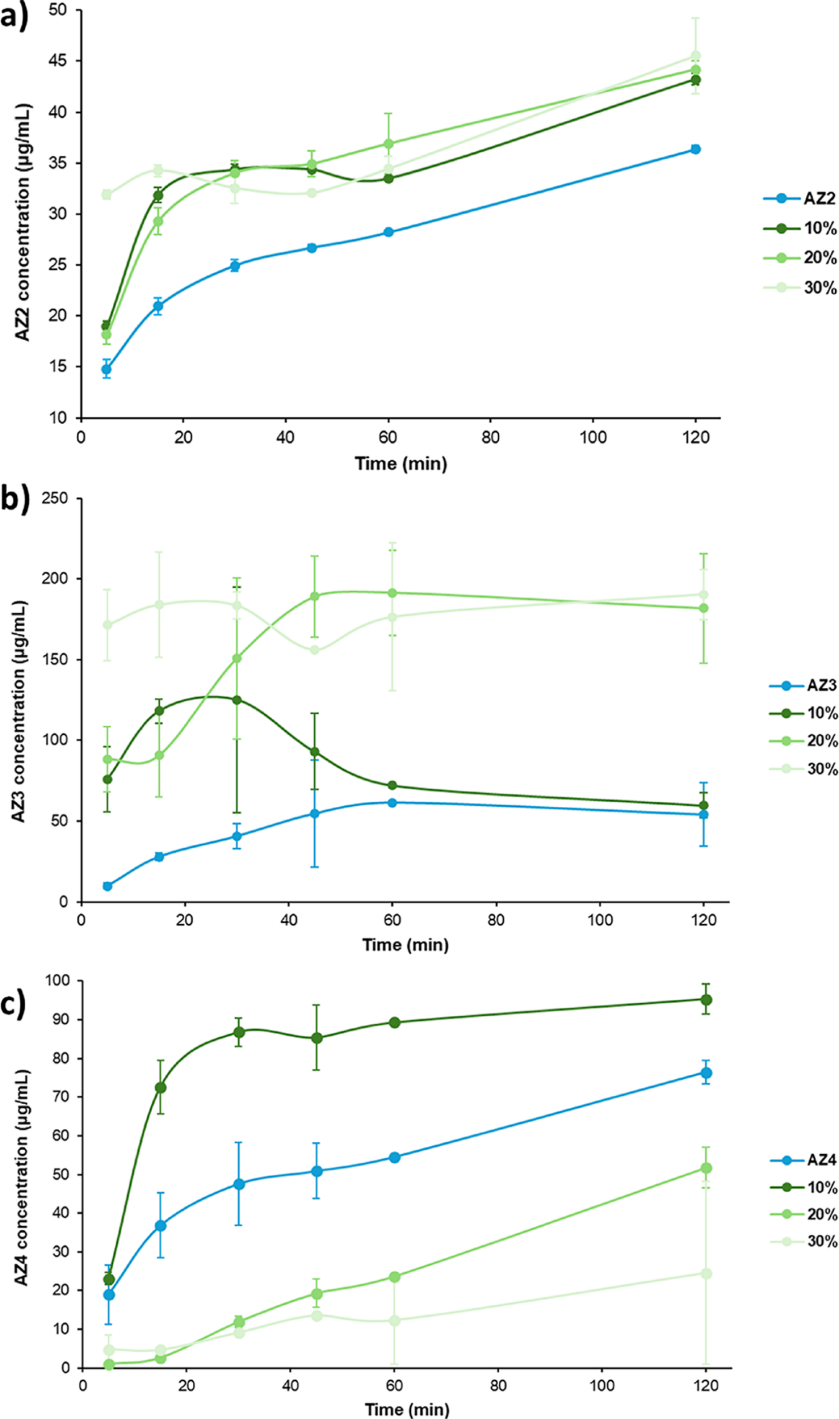
Nonsink dissolution profiles of amorphous PROTACs and HPMCAS ASDs of (a) AZ2, (b) AZ3, and (c) AZ4 prepared from 10% to 30% DL by slurry conversion, over 2 h in FaSSIF at 37 °C, and using a 10-fold nonsink condition compared to the amorphous solubilities of each PROTAC. Dissolution experiments were run in duplicate, with average concentrations plotted and error bars showing the range of concentrations measured at each time point.

Overall, the dissolution advantage provided by the ASD formulations for AZ2–4 was much more significant than for AZ1, and this formulation approach appears to be effective for increasing the kinetic solubility of this type of PROTAC compound in general, despite the ASD particles being considerably larger than the pure drug powders. ASD formulations may be able to provide some dissolution/solubility benefit for PROTAC compounds, but the design of the ASD is crucially important, and the relative level of dissolution enhancement is modest compared to more conventional drugs.^57,58^ However, further improvements, such as hierarchical ASD formulation, where the particle surfaces are modified with an additional polymer coating, may lead to even greater dissolution behavior and improved wettability.^59,60^ This strategy may be particularly effective if achieved via the slurry conversion method of ASD preparation, which has been shown here to produce ASDs of superior dissolution capability. The data presented here are also not always fully consistent with the current LoC theory (noting, e.g., the behavior of ASDs of AZ2 and AZ3), indicating that other mechanisms may be involved. This highlights the technical formulation challenge presented by the PROTAC compounds.

### ASD Conditioning

The physical stabilities of all ASDs that provided a dissolution improvement compared to the pure drug substance were assessed by XRPD and thermal analysis after conditioning the samples for 1 month at 75% RH and 40 °C, simulating a storage environment that may accelerate drug recrystallization. XRPD (ESI Figure S24) shows that no ASD samples begin to recrystallize over this time period, which is particularly of interest for AZ1, which is known to crystallize under certain conditions.^38^ The *T*_g_ values measured by a single-ramp mDSC protocol (Table 1) also showed very little deviation from the preconditioned samples, indicating that any plasticization of the ASDs by water within the humid conditioning environment does not have a significant effect on molecular mobility. TGA analysis (ESI Figure S25) shows that most of the conditioned ASDs lost between 0.1% and 0.7% weight up to 100 °C, suggesting a relatively low quantity of adsorbed moisture, followed by gradual mass loss of up to 1% between 100 °C and 180 °C as the samples exceeded *T*_g_. AZ3 ASDs at 10% and 20% DL and AZ2 ASD at 20% DL were slightly wetter than the others, with around 0.5% greater mass loss up to 100 °C compared to the other samples, and the AZ4 ASD at 10% DL contained considerably more residual solvent at approximately 1.5%. However, the generally low moisture uptake of the conditioned samples is commensurate with HPMCAS ASDs typically showing less hygroscopicity than other polymeric carriers used in the pharmaceutical industry,^61^ and suggests that the ASDs shown to provide a dissolution enhancement in this study are likely to be stable against plasticization and phase separation for months.

**Table 1:** *T*_g_ Values of ASD Samples before and after Conditioning for 1 Month at 75% RH and 40 °C^a^

sample	preconditioned *T*_g_ (°C)	conditioned *T*_g_ (°C)
AZ1 HPMCAS E 10%	129	130
AZ1 HPMCAS SC 10%	129	129
AZ1 HPMCAS SC 20%	132	128
AZ2 10%	131	132
AZ2 20%	134	139
AZ2 30%	147	147
AZ3 10%	131	132
AZ3 20%	140	139
AZ3 30%	144	144
AZ4 10%	131	131

a “E” and “SC” refer to the evaporation and slurry conversion methods of ASD preparation, respectively. All ASDs of AZ2, AZ3, and AZ4 were prepared by slurry conversion.

## Conclusions

In this work, we show that aqueous dissolution can be enhanced for a CRBN PROTAC using HPMCAS as a carrier polymer up to a drug loading of 20% w/w, whereas dissolution is not improved by using PVPVA, indicating the importance of ASD polymer selection. We have also shown that the slurry conversion method produces PROTAC ASDs with a higher limit of congruency and better dissolution performance compared to those of the solvent evaporation method. Coamorphous formulations using low-molecular-weight coformers do not show any dissolution improvement by comparison, despite spectral evidence of drug–coformer hydrogen bonds. While such interactions are not detected in the ASDs, positive deviations in measured *T*_g_ values compared to predicted values suggest that strong drug–polymer interactions may be present and could be dispersive in nature. Despite these strong interactions, the limits of congruency were higher than 30% w/w in some cases, suggesting that additional mechanisms may be involved in the dissolution behavior of these compounds compared to conventional poorly soluble drugs. The most effective formulation approach of preparing ASDs using HPMCAS as a carrier polymer via the slurry conversion method was applied successfully to three other CRBN PROTAC compounds, showing even greater dissolution enhancements compared with the pure amorphous drugs and proving the general application of the formulation strategy. All ASD samples are stable against plasticization and phase separation after 1 month of storage under elevated humidity and temperature. This work reveals that while formulation approaches using small molecule excipients may not be as suitable for enhancing the dissolution of PROTACs as they are for conventional small molecule drugs, ASD formulations prepared using an informed polymer selection and manufacturing method may be a robust approach for the pharmaceutical industry to produce commercializable solid forms of PROTACs for oral dosage units. It is also evident that general rules and understanding from ASD formulation of conventional drugs, such as the relationship between drug loading and LoC, do not necessarily apply to PROTAC ASDs and that screening must be performed for more PROTACs before general principles can be developed.

## Supplementary Material



## Data Availability

Underlying research data is available at DOI: 10.15128/r15m60qr98j.

## Notes

XRPD patterns, modulated DSC thermograms, TGA thermograms, SEM images, predicted *T*_g_ values and FTIR spectra (PDF)

## Abbreviations

PROTAC: proteolysis targeting chimera
bRo5: beyond-rule-of-5
API: active pharmaceutical ingredient
GRAS: generally recognized as safe
ASD: amorphous solid dispersion
DL: drug loading
LoC: limit of congruency
PAA: poly­(acrylic acid)
HPMCAS: hydroxypropyl methyl cellulose acetate succinate
CRBN: cereblon
PVA: poly­(vinyl alcohol)
PVPVA: polyvinylpyrrolidone vinyl acetate
XRPD: X-ray powder diffraction
DSC: differential scanning calorimetry
TGA: thermogravimetric analysis
SEM: scanning electron microscopy
UPLC: ultra performance liquid chromatography
FaSSIF: fasted state simulated intestinal fluid
RH: relative humidity
ER: estrogen receptor
LAG: liquid-assisted grinding
KGA: α-ketoglutaric acid
NBA: *m*-nitrobenzoic acid
OXA: oxalic acid
XPS: X-ray photoelectron spectroscopy.
