# Supplementary material for: Developing Multi-Component Solid Formulation Strategies for PROTAC Dissolution Enhancement
Source: Mol Pharm. 2025 Oct 2;22(11):7052–67. doi: 10.1021/acs.molpharmaceut.5c01107 (PMC12587386; doi:10.1021/acs.molpharmaceut.5c01107)
Supplement: Supplementary file 1 [file mp5c01107_si_001.pdf]

SUPPLEMENTARY INFORMATION

Developing Multi-Component Solid Formulation Strategies for PROTAC Dissolution Enhancement

Martin A. Screen,[1] Sean Askin,[2] James. F. McCabe,[3] Esther Jacobs,[2] Akosua Anane-Adjei,[2] Clare S. Mahon,[1] Mark R. Wilson,[1] Jonathan W. Steed[1,*]

[1]Durham University, Department of Chemistry, South Road, Durham DH1 3LE, United Kingdom; [2]Advanced Drug Delivery, Pharmaceutical Sciences, R&D, AstraZeneca, Cambridge CB2 0AA, United Kingdom. [3]Early Pharmaceutical Development & Manufacture, Pharmaceutical Sciences, R&D, AstraZeneca, Macclesfield SK10 2NA, United Kingdom; *Tel: +44 191 334 2085; Email: jon.steed@durham.ac.uk

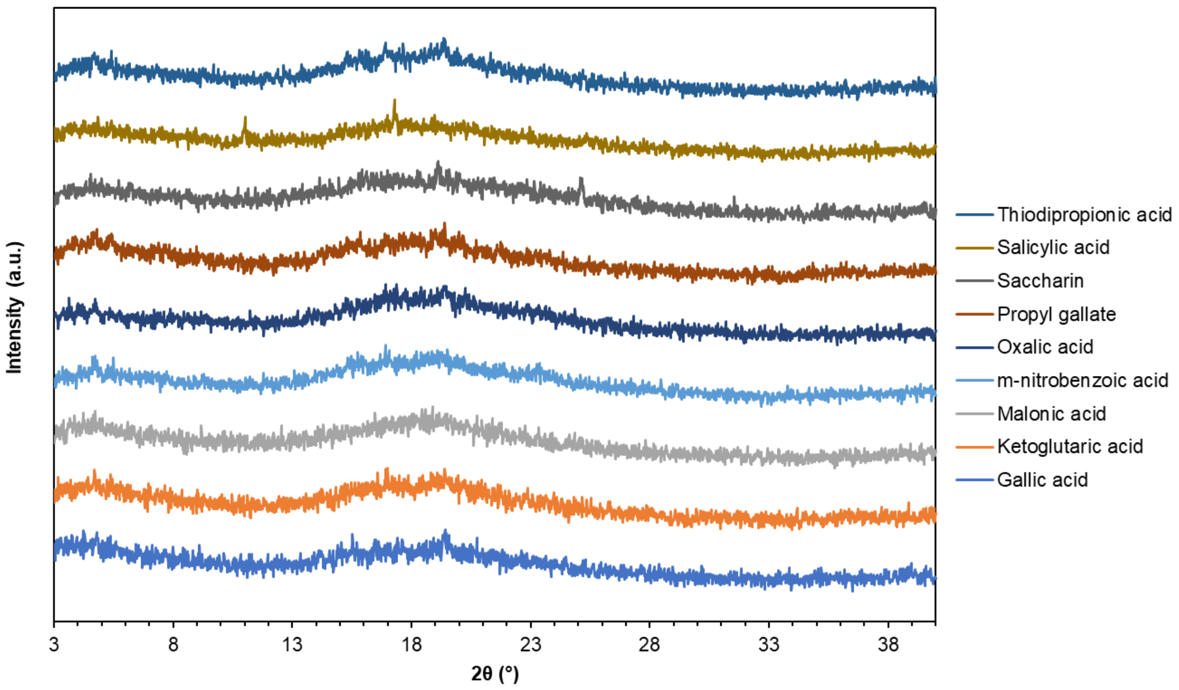

Figure S1. XRPD patterns of all nine potential co-amorphous solids showing a broad amorphous halo.

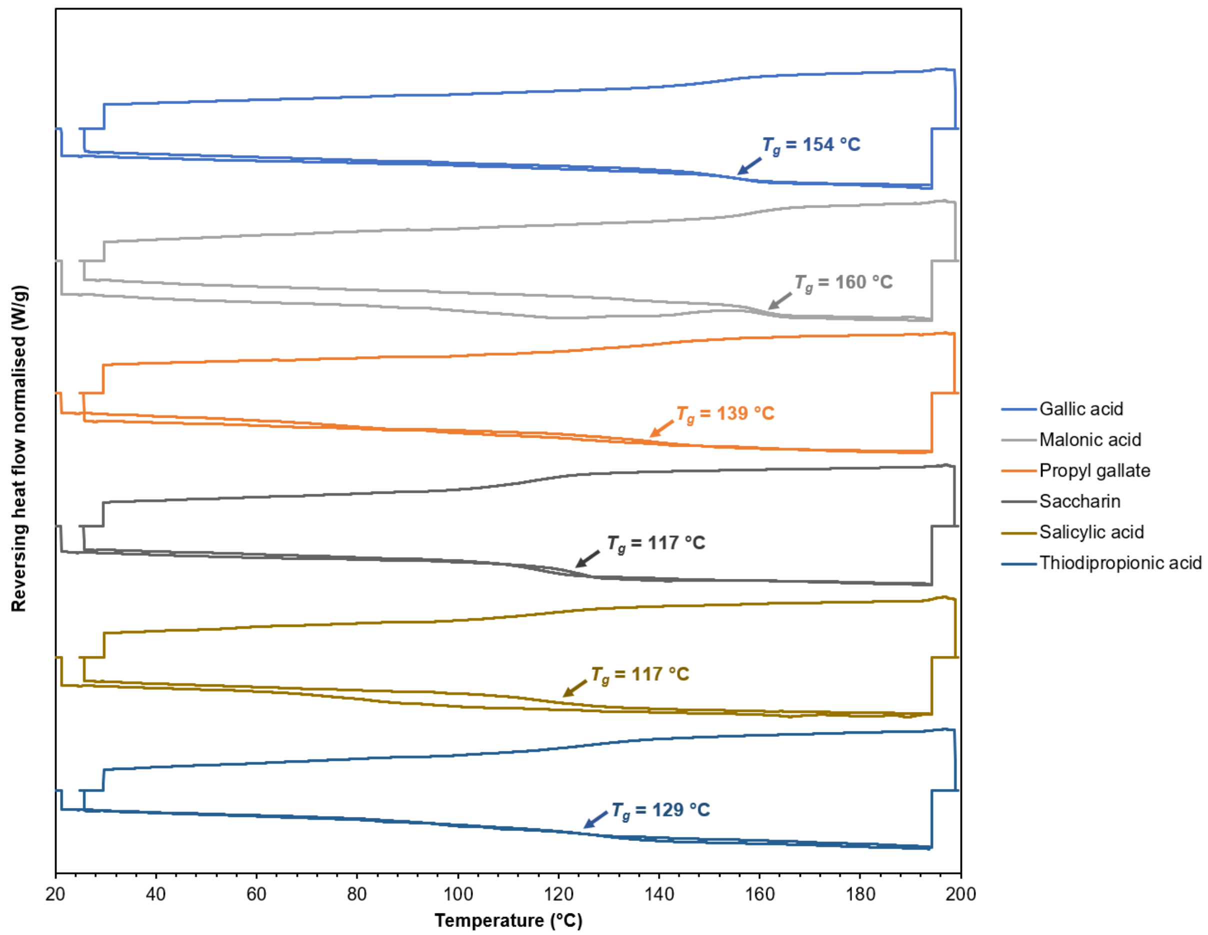

Figure S2. Heat-cool-heat mDSC thermograms for six of the nine potential co-amorphous solids not taken forward for dissolution studies, all showing a single T_g. The "dry" T_g values from the second heating cycle are marked on the plot.

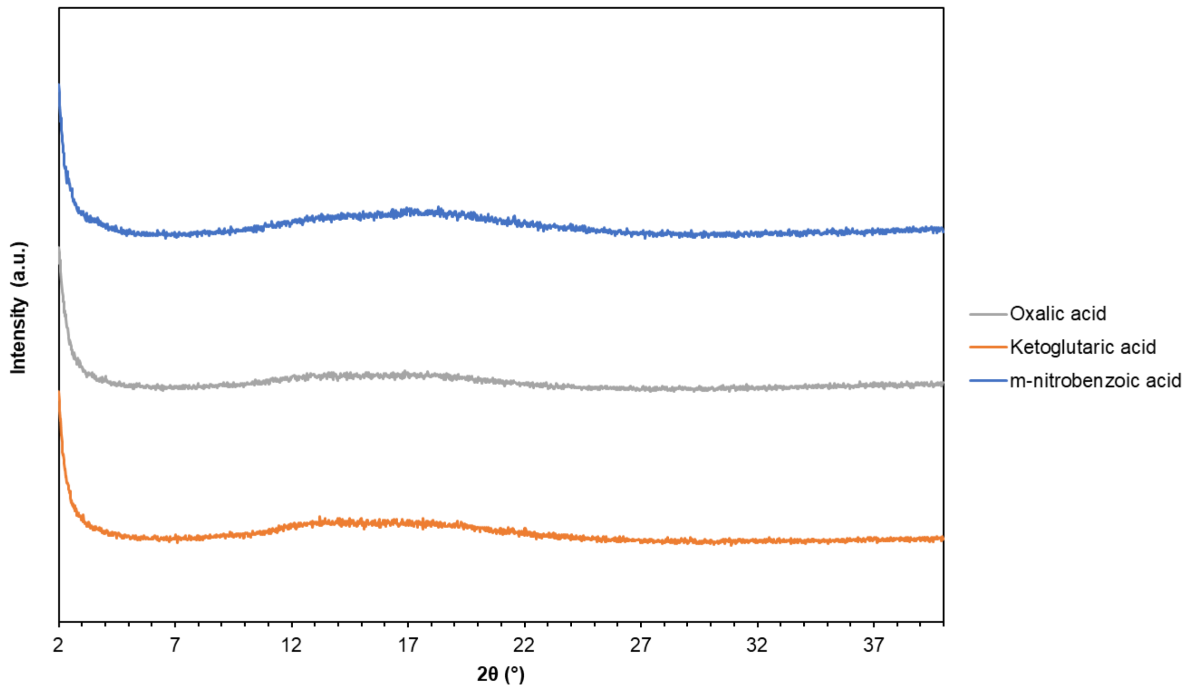

Figure S3. XRPD patterns of the three scaled-up co-amorphous solids of AZ1 with OXA, KGA and NBA showing physical stability of the amorphous phase after 3 months.

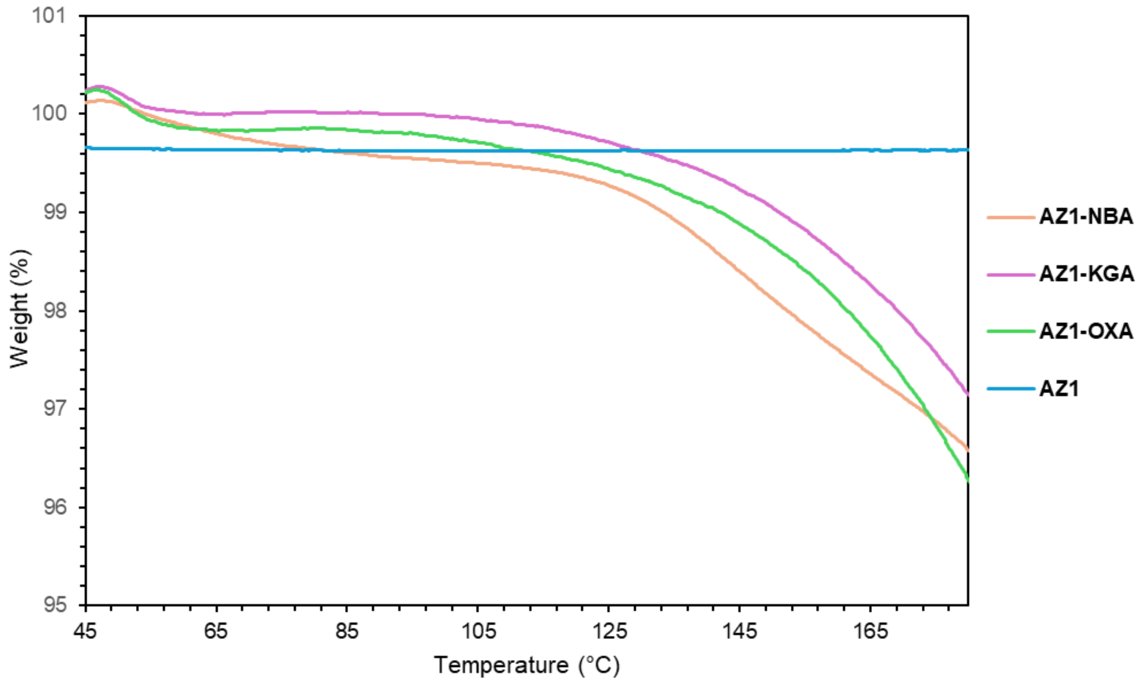

Figure S4. TGA thermograms for the co-amorphous solids of AZ1 with OXA, KGA and NBA.

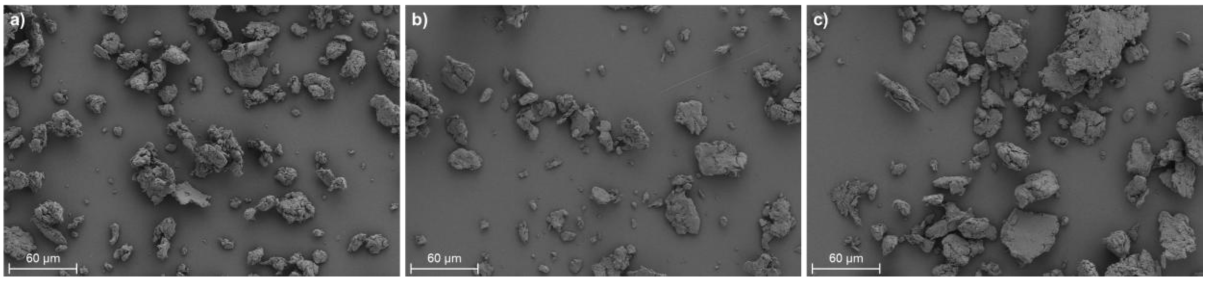

Figure S5. SEM images of co-amorphous solids of AZ1 with a) OXA, b) NBA and c) KGA.

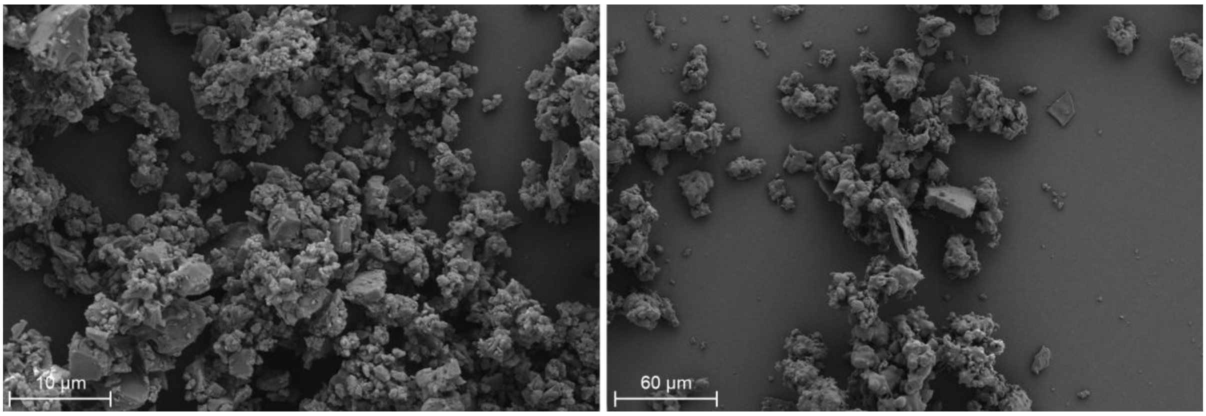

Figure S6. SEM images of milled amorphous AZ1 a) before and b) after 24 hours of slurry in FaSSIF.

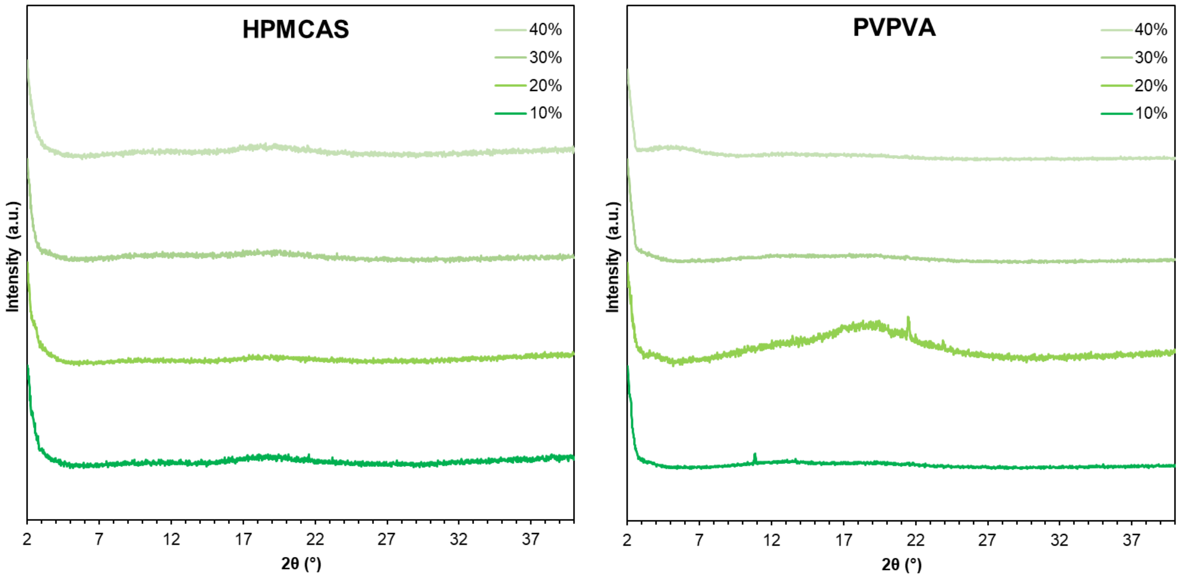

Figure S7. XRPD patterns of AZ1 ASDs with HPMCAS and PVPVA from 10 – 40 % DL prepared by slurry conversion, all showing a broad amorphous halo.

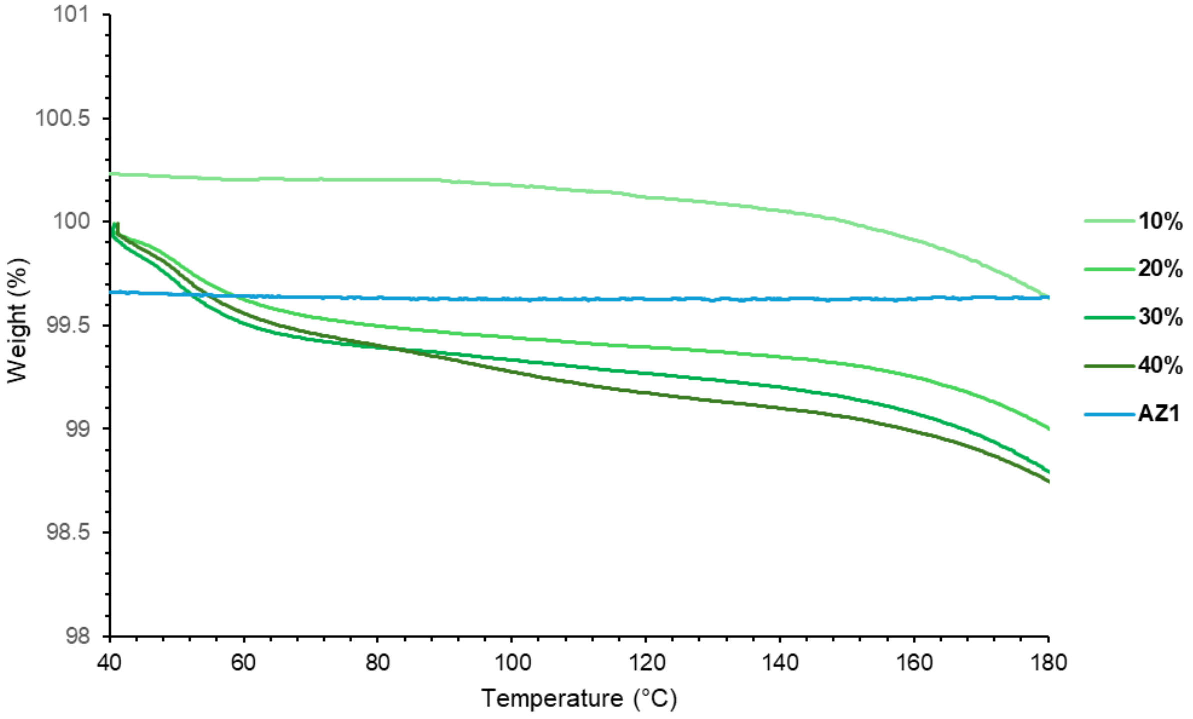

Figure S8. TGA thermograms of AZ1 HPMCAS ASDs prepared by slurry conversion from 10 – 40 % DL.

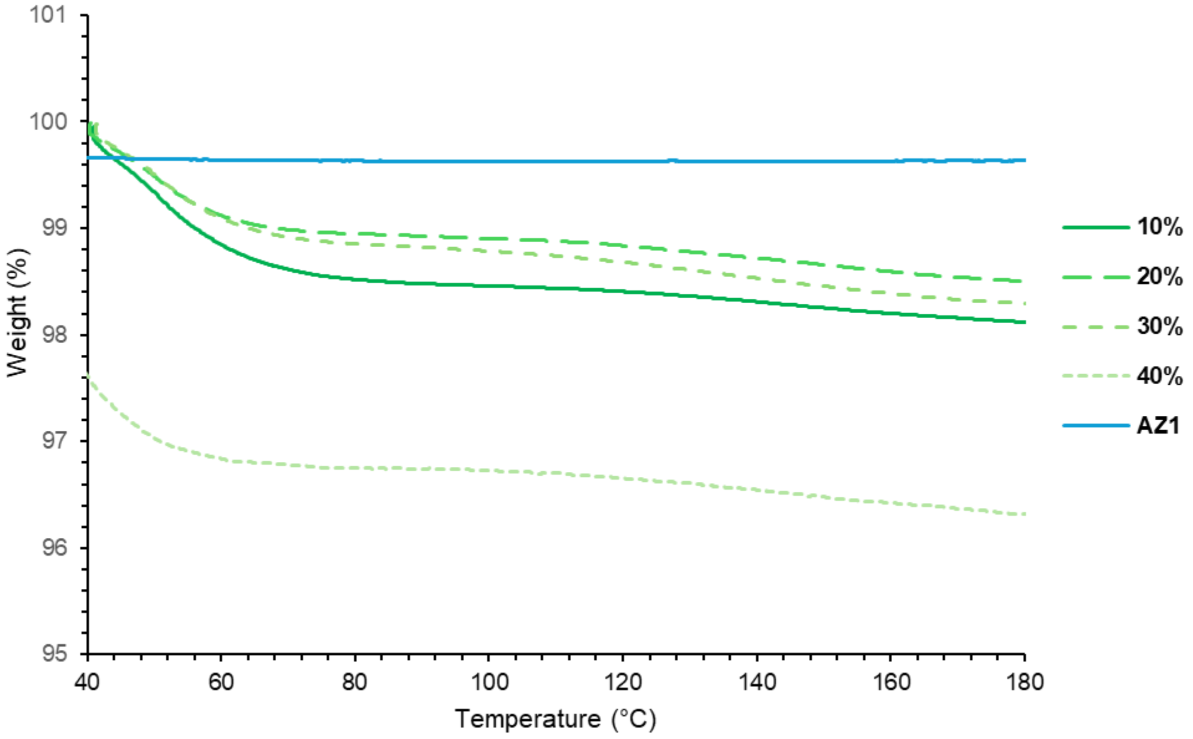

Figure S9. TGA thermograms of AZ1 PVPVA ASDs prepared by slurry conversion from 10 – 40 % DL.

Table **S1**. Comparison of experimental T_g values for AZ1 ASDs and predicted values from the Gordon-Taylor equation simplified to the Fox relation.

**PROTAC**	**Polymer**	**Method**	**DL (% w/w)**	**Measured Tg (°C)**	**Predicted Tg (°C)**	**Difference (°C)**
AZ1	HPMCAS	Slurry conversion	10	129	125	+4
			20	132	128	+4
			30	135	131	+4
			40	135	135	±0
		Solvent evaporation	10	129	125	+4
			20	135	128	+7
			30	136	131	+5
			40	139	135	+4
	PVPVA	Slurry conversion	10	115	115	±0
			20	116	118	-2
			30	121	122	-1
			40	125	126	-1
		Solvent evaporation	10	112	115	-3
			20	119	118	+1
			30	128	122	+6
			40	134	126	+8
AZ2	HPMCAS	Slurry conversion	10	131	126	+5
			20	134	129	+5
			30	147	133	+14
AZ3	HPMCAS	Slurry conversion	10	131	126	+5
			20	140	129	+11
			30	144	133	+11
AZ4	HPMCAS	Slurry conversion	10	131	126	+5
			20	140	129	+11
			30	143	133	+10

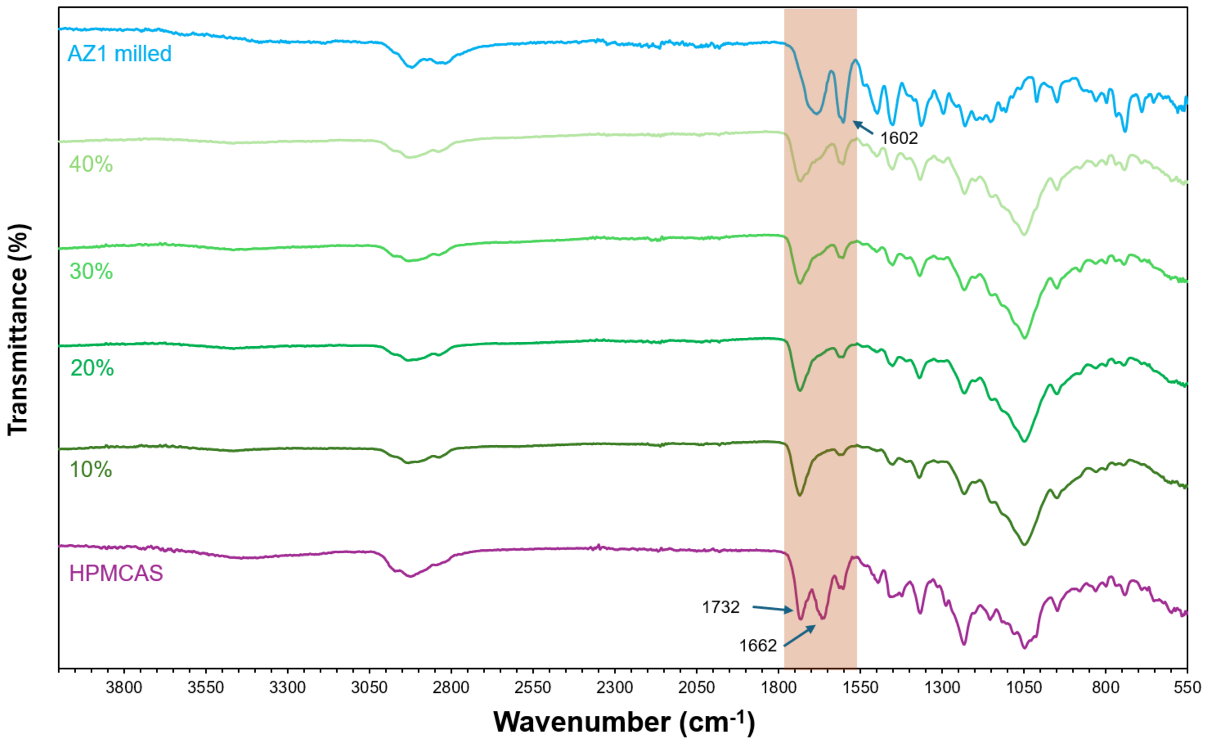

Figure S10. FTIR spectra of AZ1 HPMCAS ASDs prepared by slurry conversion from 10 – 40 % DL.

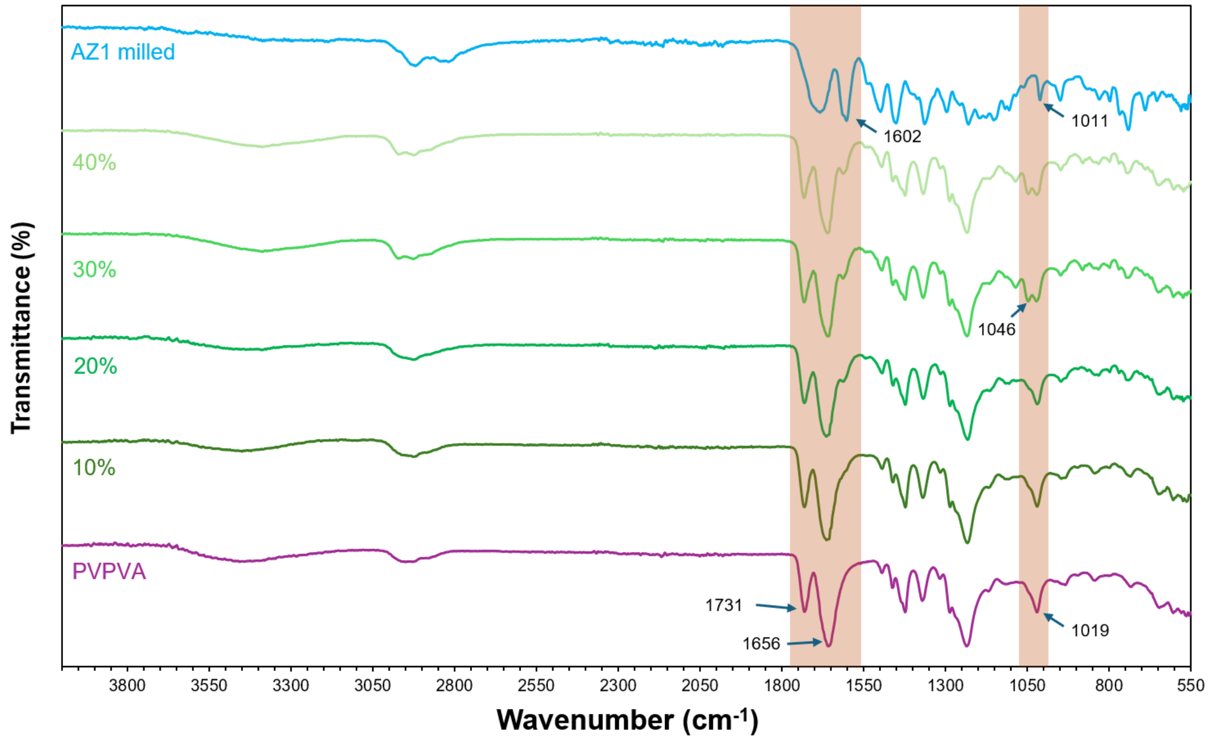

Figure S11. FTIR spectra of AZ1 PVPVA ASDs prepared by slurry conversion from 10 – 40 % DL.

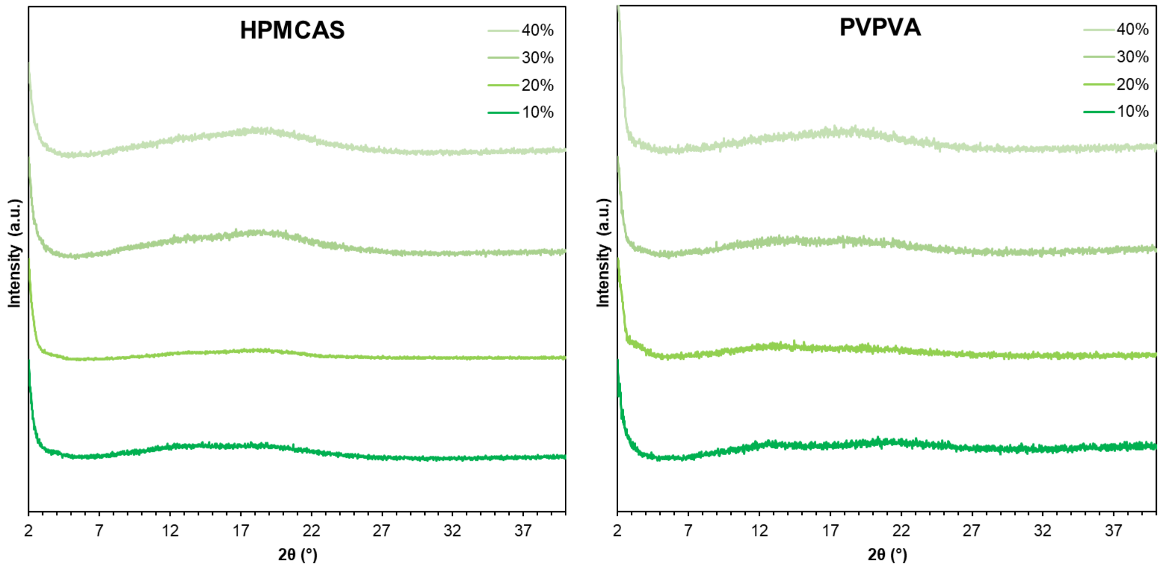

Figure S12. XRPD patterns of AZ1 ASDs with HPMCAS and PVPVA from 10 – 40 % DL prepared by solvent evaporation, all showing a broad amorphous halo.

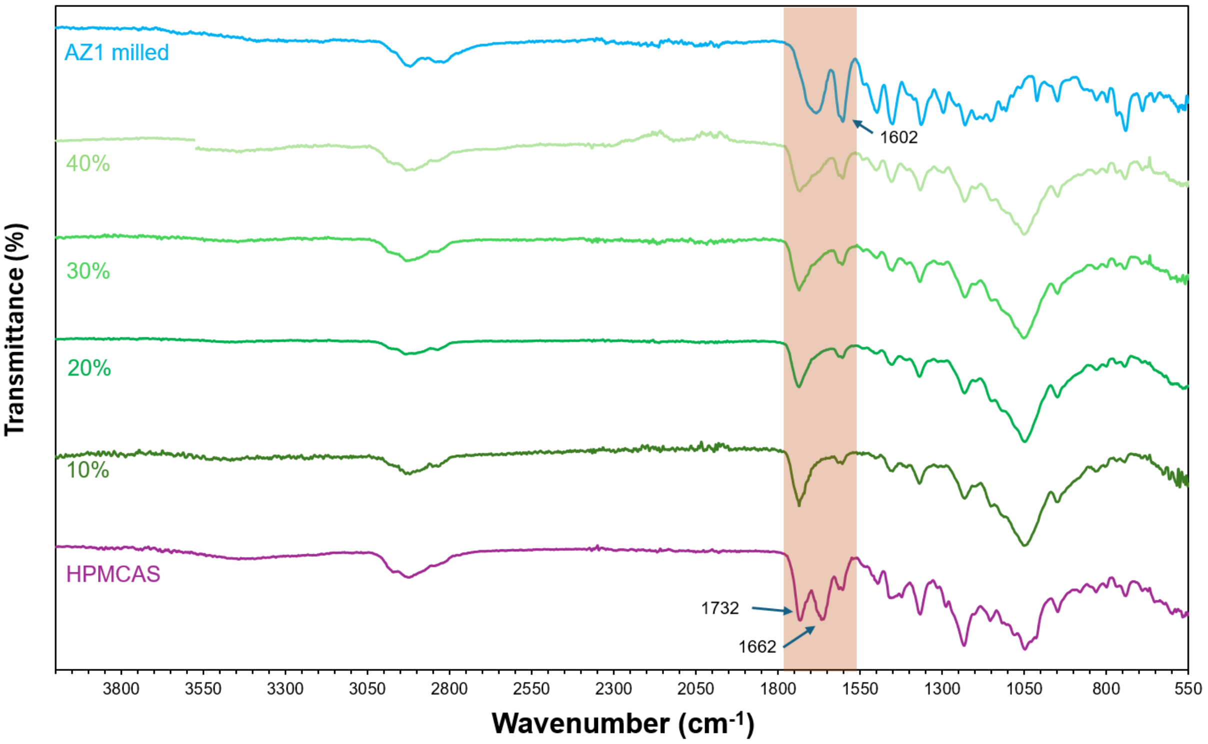

Figure S13. FTIR spectra of AZ1 HPMCAS ASDs prepared by solvent evaporation from 10 – 40 % DL.

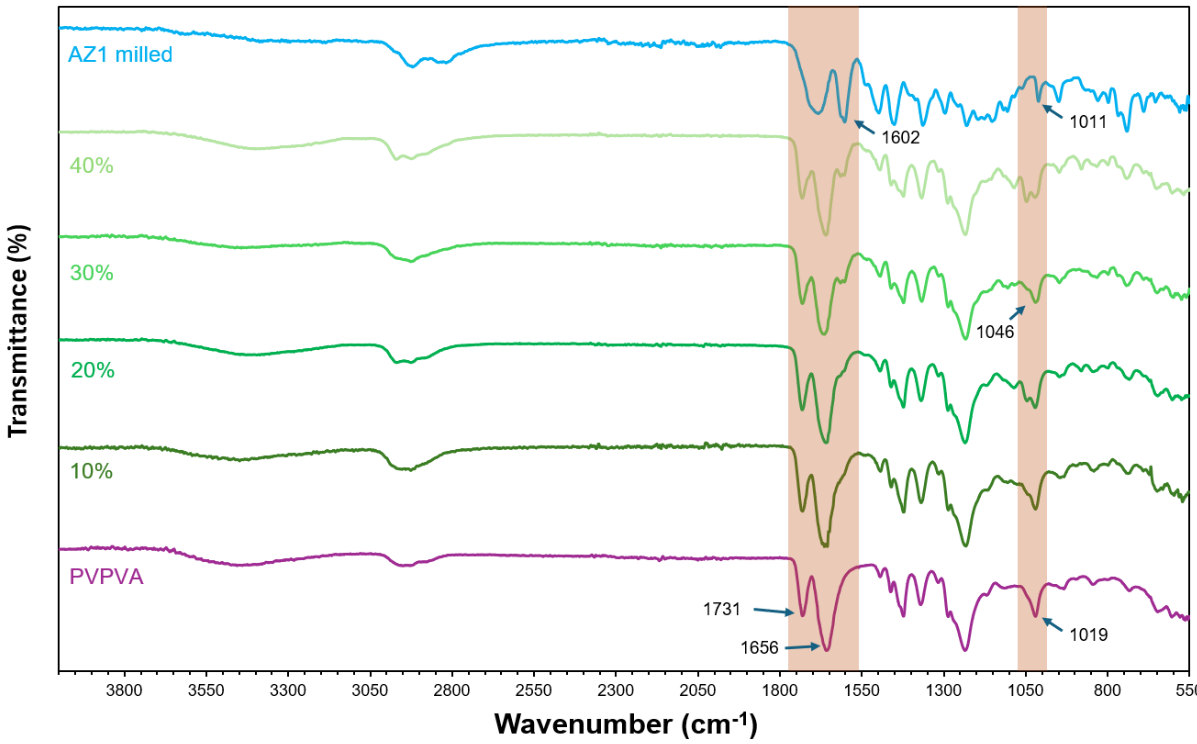

Figure S14. FTIR spectra of AZ1 PVPVA ASDs prepared by solvent evaporation from 10 – 40 % DL.

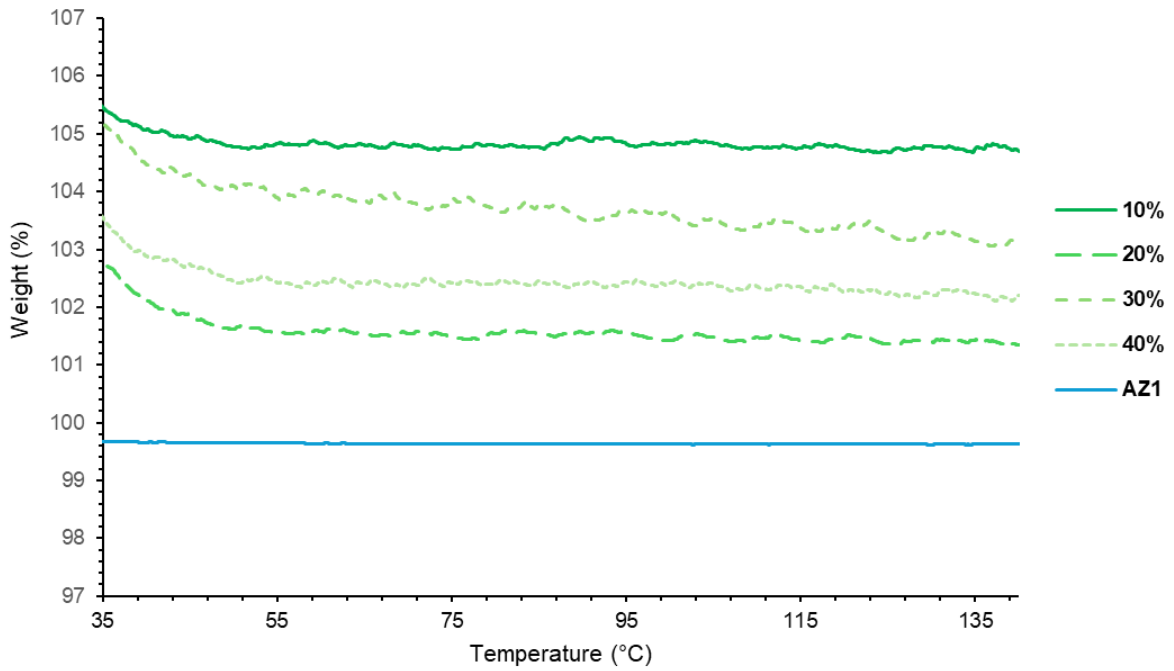

Figure S15. TGA thermograms of AZ1 HPMCAS ASDs prepared by solvent evaporation from 10 – 40 % DL.

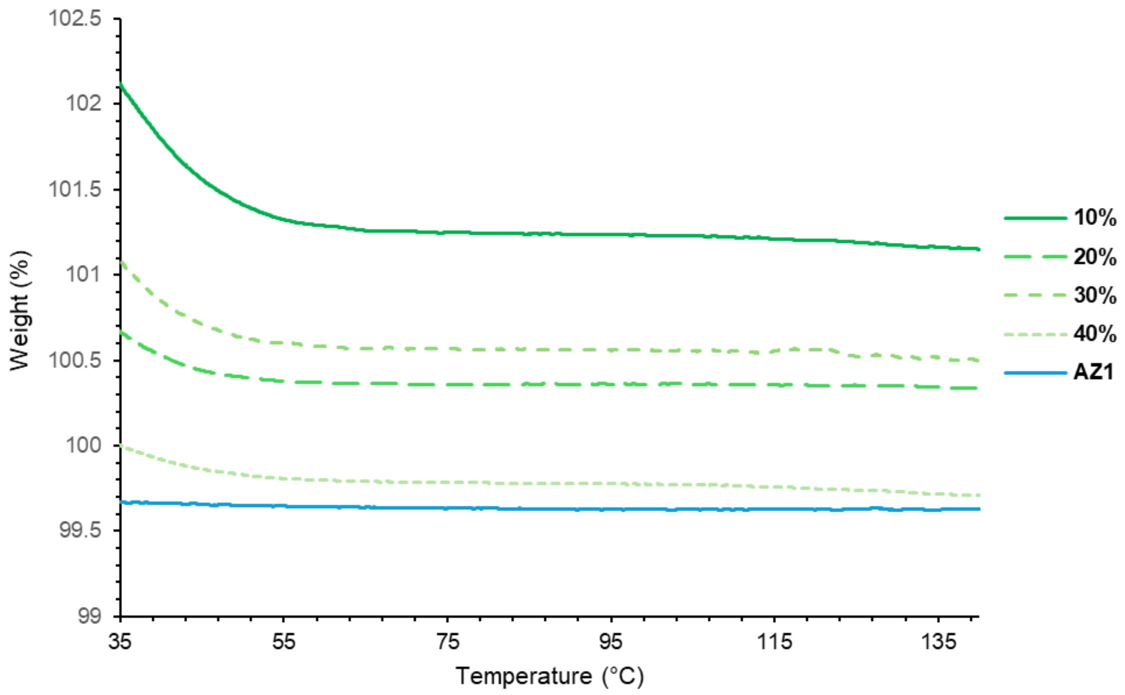

Figure S16. TGA thermograms of AZ1 PVPVA ASDs prepared by solvent evaporation from 10 – 40 % DL.

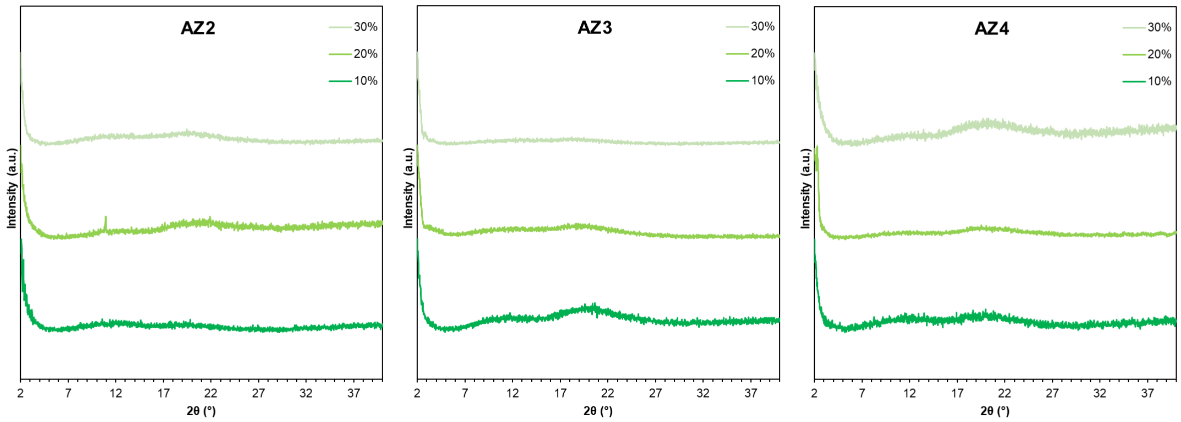

Figure S17. XRPD patterns for AZ2, AZ3 and AZ4 ASDs with HPMCAS from 10 – 30 % DL prepared by slurry conversion, all showing a broad amorphous halo.

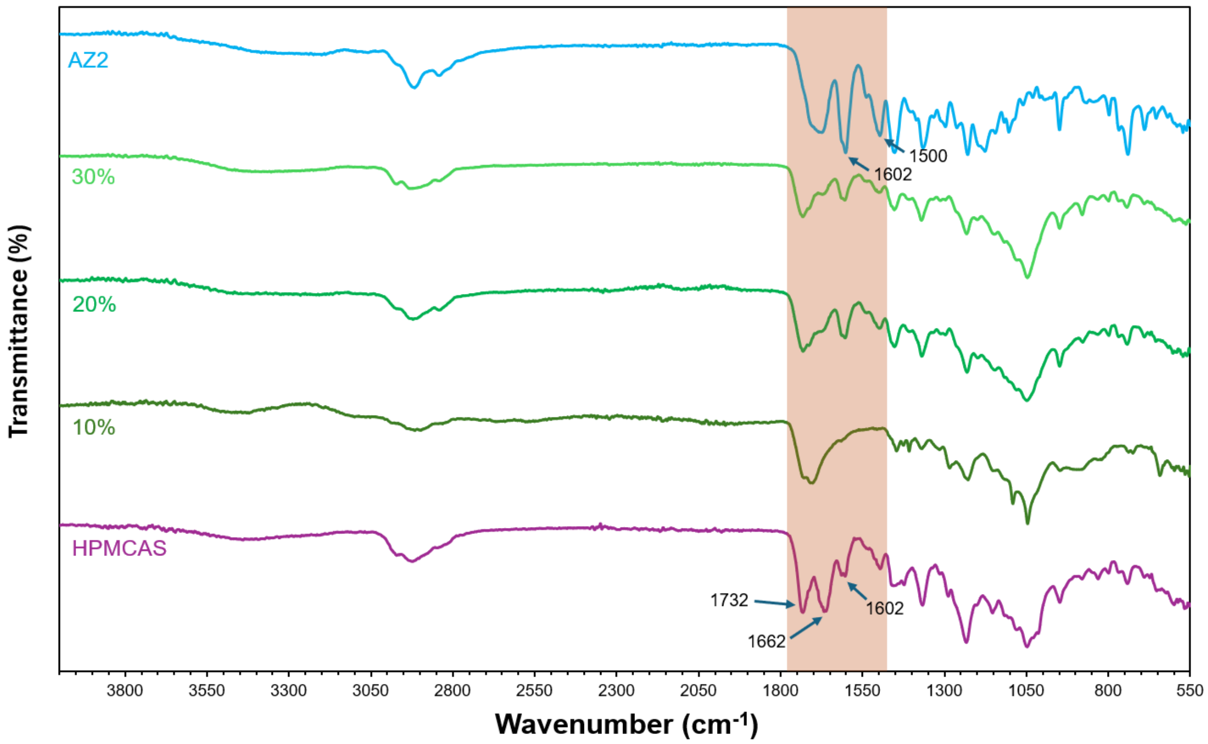

Figure S18. FTIR spectra of AZ2 HPMCAS ASDs from 10 – 30 % DL.

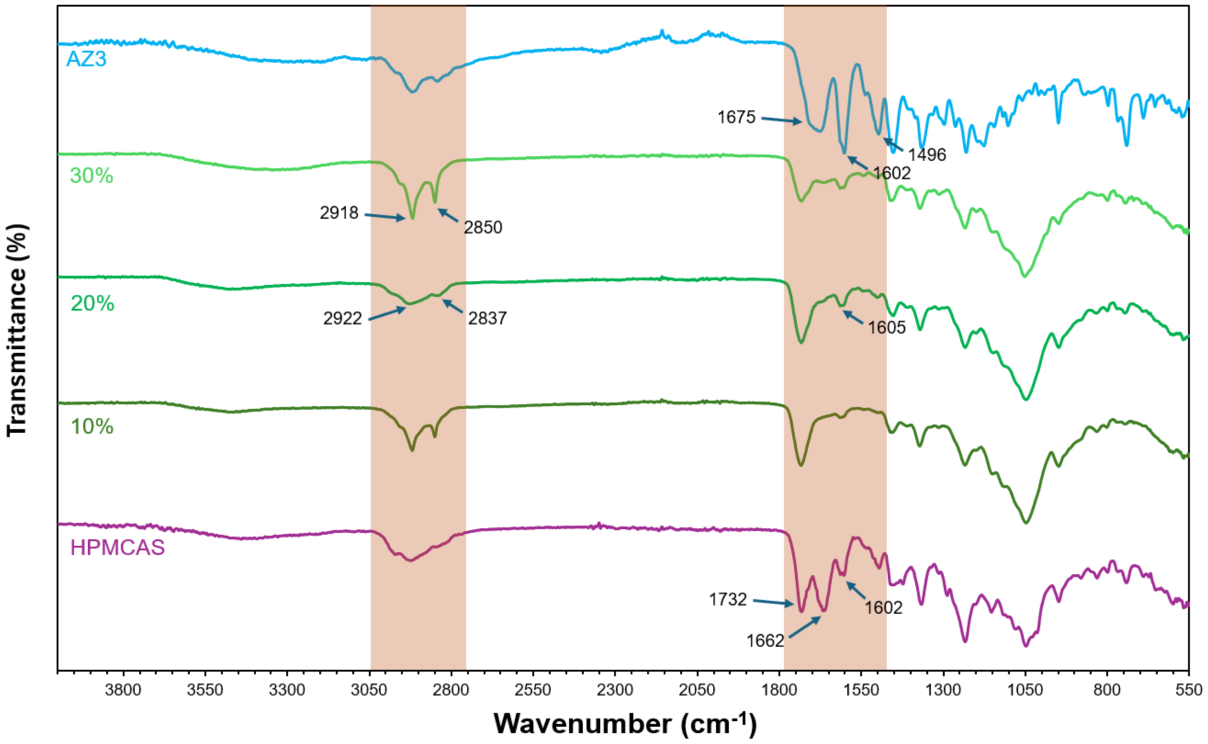

Figure S19. FTIR spectra of AZ3 HPMCAS ASDs from 10 – 30 % DL.

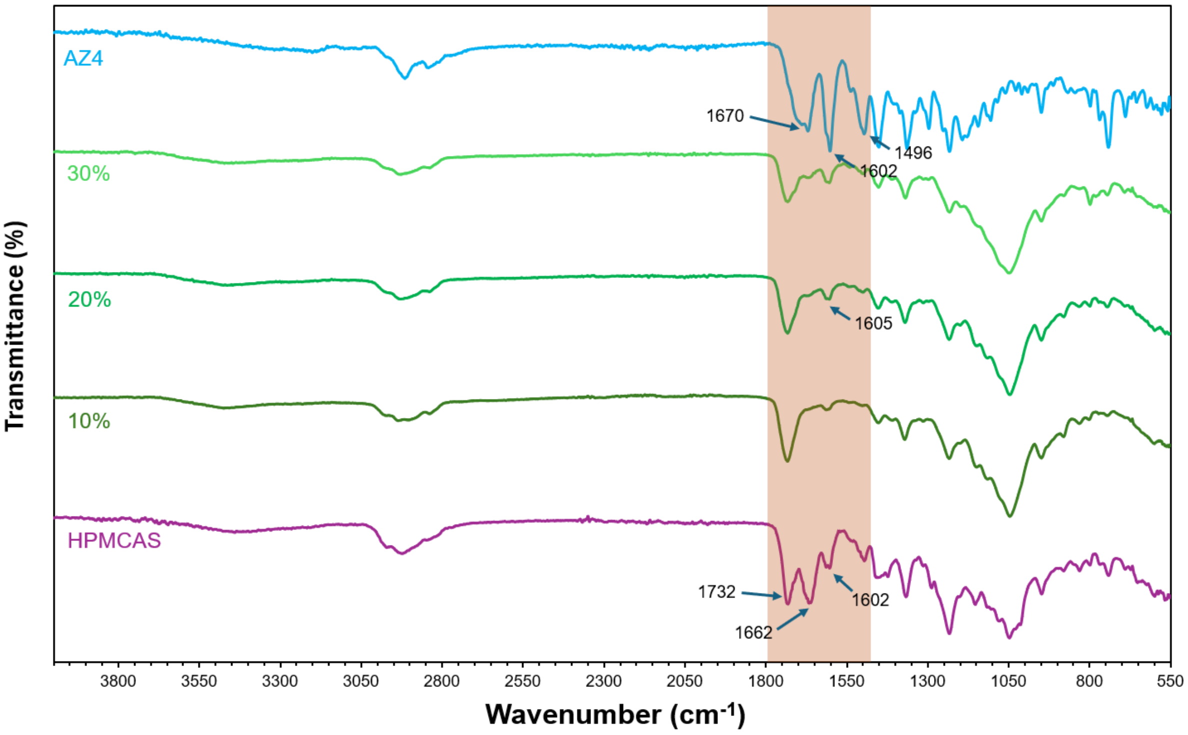

Figure S20. FTIR spectra of AZ4 HPMCAS ASDs from 10 – 30 % DL.

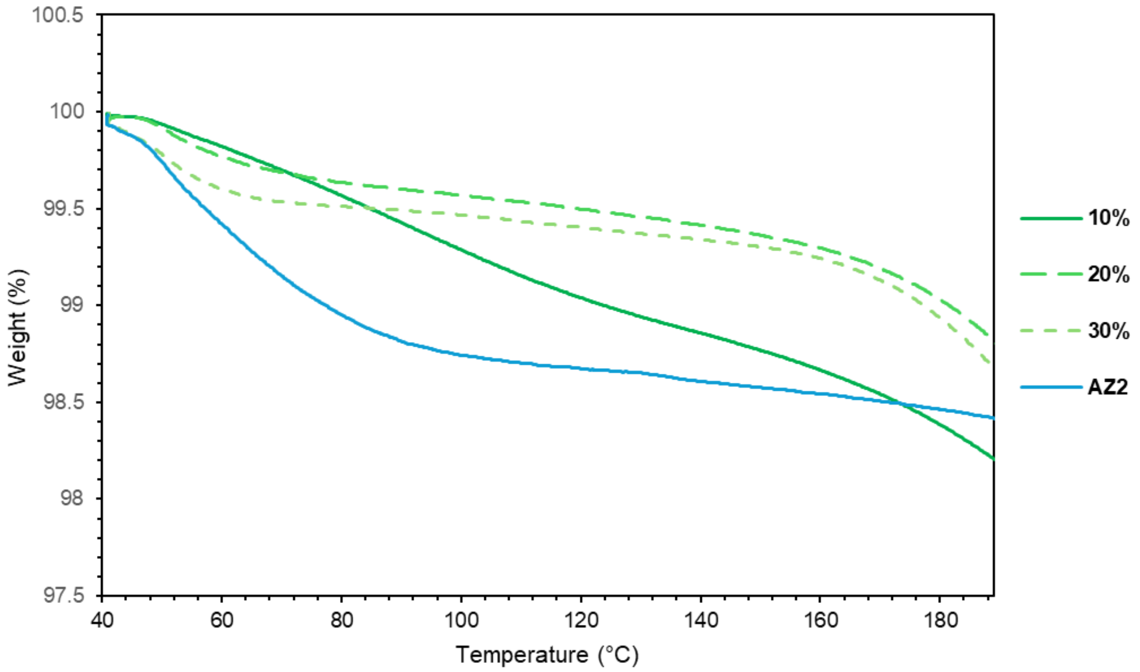

Figure S21. TGA thermograms of AZ2 HPMCAS ASDs from 10 – 30 % DL. AZ2 contains roughly 1.3 % w/w of water.

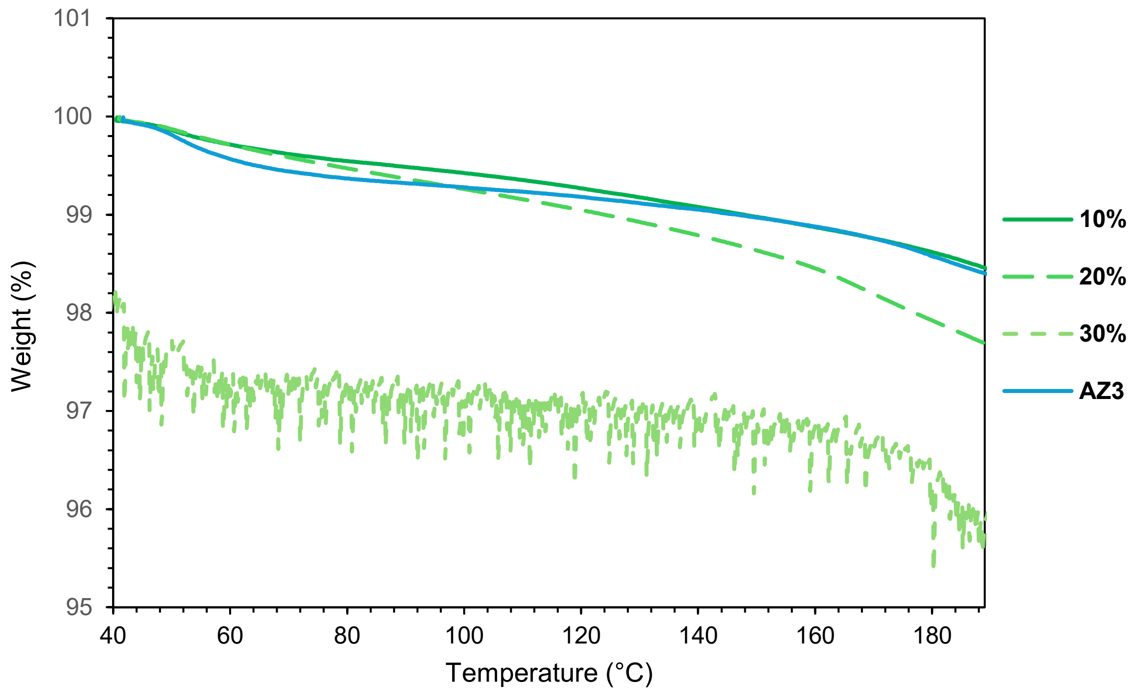

Figure S22. TGA thermograms of AZ3 HPMCAS ASDs from 10 – 30 % DL. AZ3 contains roughly 0.7 % w/w of water.

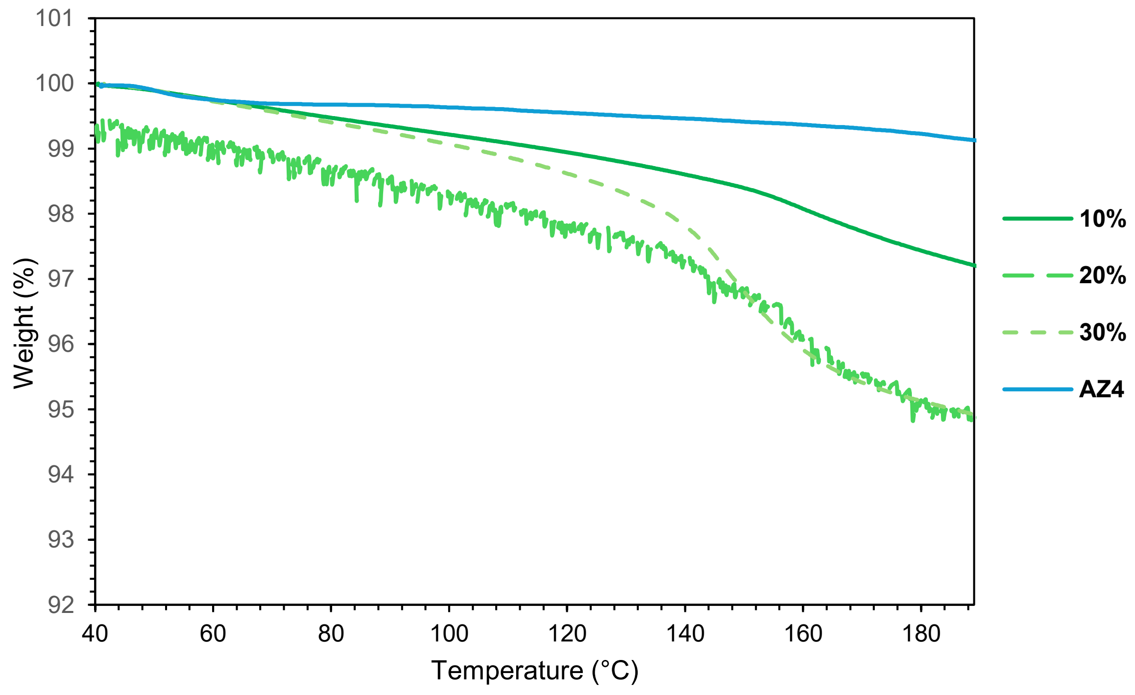

Figure S23. TGA thermograms of AZ4 HPMCAS ASDs from 10 – 30 % DL. AZ4 contains 0.4 % w/w of water.

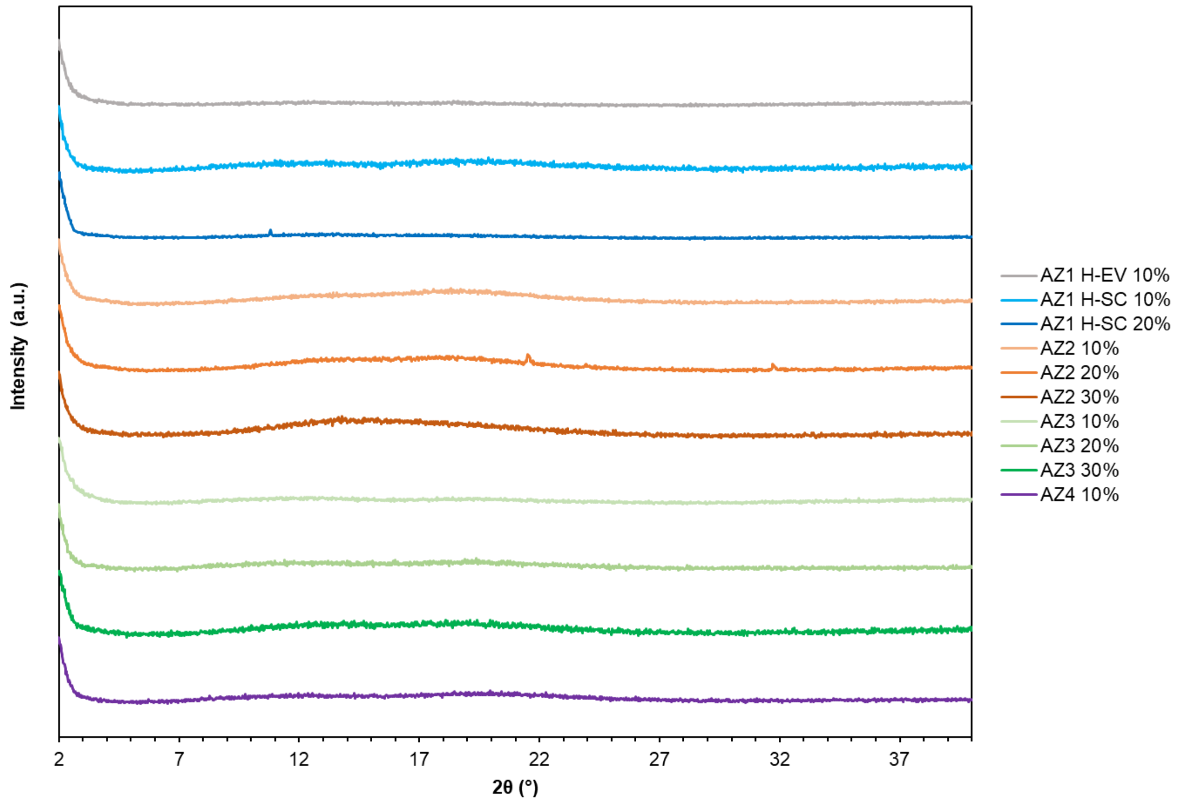

Figure S24. XRPD patterns for ASDs conditioned for 1 month at 75% RH and 40 °C, all showing a broad amorphous halo.

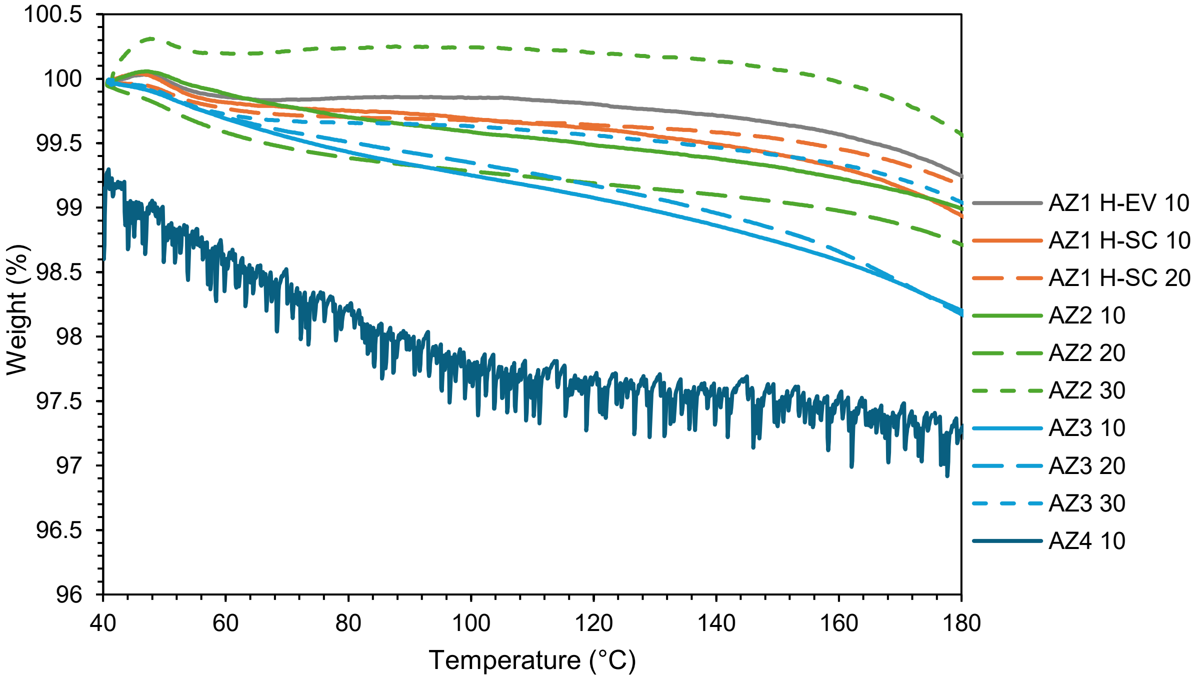

Figure S25. TGA thermograms for ASDs conditioned for 1 month at 75% RH and 40 °C.

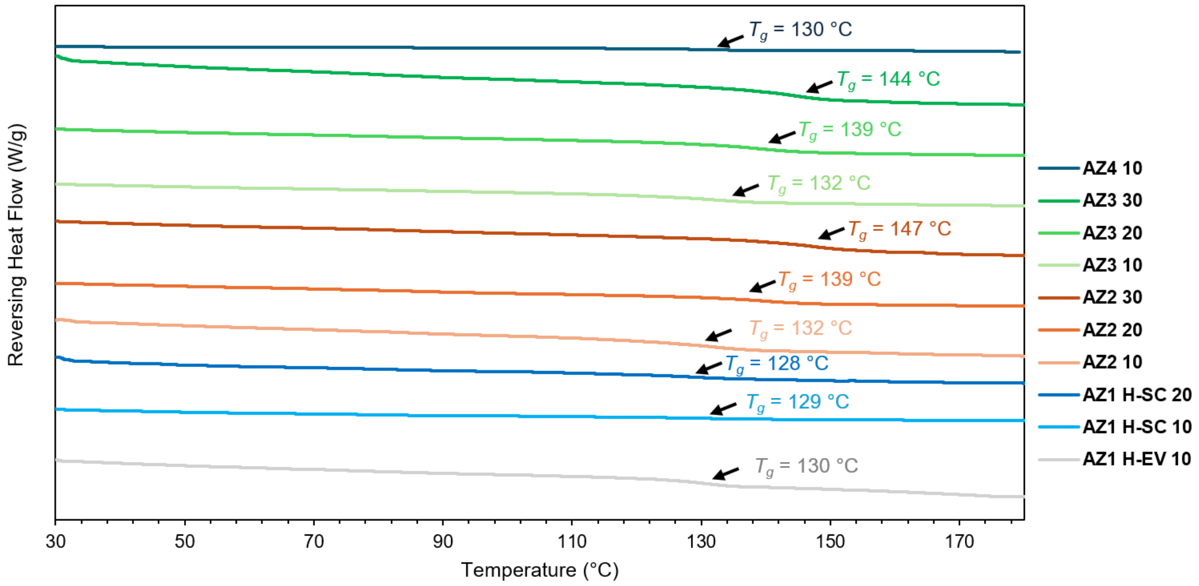

Figure S26. mDSC thermograms for ASDs conditioned for 1 month at 75% RH and 40 °C.
